# Incidence of hospitalization for infection among patients with hepatitis B or C virus infection without cirrhosis in Taiwan: A cohort study

**DOI:** 10.1371/journal.pmed.1002894

**Published:** 2019-09-13

**Authors:** Yen-Chieh Lee, Jiun-Ling Wang, Yaa-Hui Dong, Hsi-Chieh Chen, Li-Chiu Wu, Chia-Hsuin Chang

**Affiliations:** 1 Department of Family Medicine, Cathay General Hospital, Taipei, Taiwan; 2 Department of Medicine, College of Medicine, Fu Jen Catholic University, Taipei, Taiwan; 3 Department of Internal Medicine, National Cheng Kung University Hospital, Tainan, Taiwan; 4 Department of Medicine, National Cheng Kung University Medical College, Tainan, Taiwan; 5 Faculty of Pharmacy, School of Pharmaceutical Science, National Yang-Ming University, Taipei, Taiwan; 6 Institute of Public Health, School of Medicine, National Yang-Ming University, Taipei, Taiwan; 7 Institute of Epidemiology and Preventive Medicine, College of Public Health, National Taiwan University, Taipei, Taiwan; 8 Department of Internal Medicine, National Taiwan University Hospital, Department of Medicine, College of Medicine, National Taiwan University, Taipei, Taiwan; 9 Institute of Epidemiology and Preventive Medicine, College of Public Health, National Taiwan University, Taipei, Taiwan; University of Texas Southwestern Medical Center, UNITED STATES

## Abstract

**Background:**

Infection is a major complication in liver cirrhosis and causes major morbidity and mortality. However, the incidence and mortality related to these conditions in patients infected with hepatitis C virus (HCV) are unclear, as is whether antiviral therapy could change their infection risk.

**Methods and findings:**

In this community-based cohort study, a total of 115,336 adults (mean age 52.2 years; 35.6% men) without cirrhosis participating in the New Taipei City Health Screening in 2005–2008 were classified as having noncirrhotic HCV (NC-HCV) (*n* = 2,839), noncirrhotic hepatitis B virus (NC-HBV) (*n* = 8,316), or no HBV or HCV infection (NBNC) (*n* = 104,181). Participants were followed to their first hospitalization for infection or death after data linkage with the Taiwan National Health Insurance Research Database (NHIRD) and Death Registry. A Cox proportional hazard regression model, adjusted for age, sex, body mass index (BMI), smoking, alcohol consumption, education level, diabetes, renal function, systemic steroids, and history of hospitalization, was used to calculate hazard ratios (HRs) and 95% confidence intervals (CIs) for overall and individual sites of infection and infection-related mortality. The reference group was NBNC participants with normal to mildly elevated alanine aminotransferase (ALT) (<1.5 times upper normal limit [UNL]) levels. To further address the impact of antiviral treatment on infection risk, we conducted analyses of data from the nationwide NHIRD and compared the risks for hospitalization because of infections and infection-related deaths between patients with HCV who received antiviral therapy (*n* = 20,264) and those who remained untreated (*n* = 104,360). During a median 8.2-year follow-up, the incidence of hospitalization for infection was substantially higher in NC-HCV patients. Compared to the reference group, NC-HCV was associated with a significantly higher risk for hospitalization because of overall infections (adjusted HR: 1.22; 95% CI: 1.12–1.33), but we observed no increased risk for patients in the NC-HBV (adjusted HR: 0.94; 95% CI: 0.88–1.01) or NBNC group with moderate to markedly elevated ALT levels (adjusted HR: 1.03; 95% CI: 0.93–1.14). For specific sites of infection, the NC-HCV group had increased risks for septicemia and lower respiratory tract, reproductive, and urinary tract infections. We noted no increased risk for infection-related death among patients with NC-HCV. Patients with HCV who received antiviral therapy had significantly reduced infection-related hospitalization and death risks (adjusted HR: 0.79; 95% CI: 0.73–0.84 for infection-related hospitalization and adjusted HR: 0.08; 95% CI: 0.04–0.16 for infection-related deaths). Study limitations include the exclusion of patients with cirrhosis from the cohort, the possibility of unmeasured confounding, and the lack of information on direct-acting antiviral agents (DAAs).

**Conclusions:**

In this study, patients with NC-HCV were at increased risk for hospitalization for infection, while no increased risk was observed for NC-HBV-infected patients.

## Abstract 2

Chia-Hsuin Chang and co-workers study risk of hospitalization for infection in people with hepatitis B or C virus infection without cirrhosis.

## Author summary

**Why was this study done?:**

- Hepatitis B virus (HBV) and hepatitis C virus (HCV) infections are major causes of chronic hepatitis that generally result in liver cirrhosis and subsequent hepatocellular carcinoma.
- Other infections are a major complication and cause major morbidity and mortality in patients with cirrhosis.

**What did the researchers do and find?:**

- In this community-based cohort of more than 110,000 participants with 8 years of follow-up, we found that patients without cirrhosis and with HCV infections (NC-HCV), but not patients without cirrhosis and with HBV infections (NC-HBV), had a 22% increased risk for hospitalization for overall infection episodes.
- For individual sites of infection, patients with NC-HCV had increased risks for septicemia and lower respiratory tract, reproductive, and urinary tract infections.
- Patients with HCV infection who received ribavirin plus peg-interferon therapy had a 20% reduced risk for hospitalization for all infections.

**What do these findings mean?:**

- Healthcare professionals should make early diagnosis and treatment when there is clinical suspicion of infection syndrome in HCV patients.
- Further studies are needed to explore whether HCV eradication or other prevention strategies (e.g., vaccination) could reduce the risk for hospitalization for infection.

## Introduction

Hepatitis C virus (HCV) infection is a major cause of chronic hepatitis and generally results in liver cirrhosis and subsequent hepatocellular carcinoma after decades of exposure [1]. HCV infects 130 to 210 million people worldwide, and 75% to 85% of them are persistently infected after initial exposure to the virus [2]. Taiwan, as a hyperendemic area for chronic liver disease, has the highest prevalence rate for HCV infection in Northeast Asia [3]. The HCV seroprevalence in Taiwan, according to an earlier nationwide community-based survey, varies greatly among cities (ranging from 1.6% to 19.6%), and iatrogenic factors are reported to be the major routes of transmission [4].

The incidence of bacterial infection is increased in patients with cirrhosis, which can be explained by bacterial translocation and a decreased ability to clear cytokines and pathogens from the circulation [5,6]. Patients infected with HCV may be predisposed to several other infectious diseases, probably through transmission routes that these pathogens share with HCV or because of an immunocompromised state resulting from cirrhosis or hepatocellular carcinoma [7]. Several studies also suggest distinctive characteristics of HCV that cause immune dysfunction and a subsequent increased vulnerability to infections [8–12]. In one large, hospital-based case-control study among United States veterans, El-Serag and colleagues found that HCV infection was associated with a broad range of infectious diseases [7]. Other studies have shown that HCV infection is a risk or prognostic factor for various infectious diseases with respect to specific pathogens [13–17] in patients with other comorbidities who are undergoing certain procedures [18–22] or involving specific organ systems [17,23]. However, most of these investigations were hospital-based studies with small case numbers and limited control for confounding factors. To our knowledge, no study has comprehensively evaluated the overall risk for infection or particular different sites of infection among patients infected with HCV who do not have cirrhosis. Furthermore, no study has investigated the impact of antiviral therapy on infection risks in patients with chronic HCV.

Using data from a large community-based health screening program, we conducted a cohort study to examine the risk for a wide range of clinically important infectious diseases and infection-related mortality among patients infected with hepatitis B virus (HBV) or HCV and without cirrhosis (NC-HBV and NC-HCV, respectively) under the hypothesis that patients with HCV infection would have an increased risk for hospitalization for infectious disease and infection-associated mortality compared with those without HBV or HCV infection (NBNC). Finally, we compared the risks for hospitalization for infection and infection-related deaths between patients with HCV or HBV who received antiviral therapy with those who did not receive antiviral therapy.

## Methods

### Risks for infectious disease–related morbidity and mortality among patients with NC-HBV or NC-HCV

#### Data source and study population

The study is a retrospective analysis of prospectively collected data for 125,865 individuals who voluntarily participated in a free, community-based health screening service for residents aged 20 years or older in New Taipei City from March 5, 2005, to July 27, 2008. Details of the study cohort have been described previously [24]. In brief, the participants completed a questionnaire about their demographics, education level, and lifestyle. Each participant provided written informed consent and underwent a standard physical examination including anthropomorphic measurements and blood and urine analyses. Blood samples were collected after an overnight fast, and first morning–voided urine was collected and analyzed. Participants signed consent forms, and data related to individual identification were removed and remained anonymized during the entire study process.

The screening program database was linked to the National Health Insurance Research Database (NHIRD) and the National Death Registry using each participant’s unique national identification number. In Taiwan, national health insurance is compulsory for all residents, and the coverage rate is over 99% [25]. The National Taiwan University Hospital Research Ethics Committee approved the protocol. The original prospective analysis plan is available (S1 Text). The main analysis regarding the risks for infectious disease–related morbidity and mortality among NC-HBV and NC-HCV patients was consistent with the prospective analysis plan. The sensitivity analyses and the analyses exploring the effect of antiviral therapy on infectious disease–related morbidity and mortality among viral hepatitis patients were specified at the data analysis and revision stage. This study is reported according to the Strengthening the Reporting of Observational Studies in Epidemiology (STROBE) guideline (S2 Text).

Participants were excluded if they were (1) younger than 20 years old; (2) did not have a baseline measurement of viral hepatitis markers, liver function tests, serum creatinine, body mass index (BMI), and fasting glucose levels; (3) did not have complete information about cigarette smoking, alcohol consumption, and education level; (4) did not have any inpatient or outpatient records in the NHI database; or (5) had a diagnosis of liver cirrhosis identified from the diagnostic codes of the insurance database before and after the health screening program. A previous validation study based on administrative databases reported good accuracy with a positive predictive value of 90% and a negative predictive value of 87% when using the algorithm to identify cirrhosis patients [26].

#### Measurement of liver disease status and other covariates

Based on information from the baseline laboratory data, participants were classified into the following categories according to their liver function test results and viral hepatitis marker status: (1) NBNC (subclassified into patients with normal to mildly elevated liver enzyme [alanine aminotransferase (ALT) level normal to 1.5 × upper normal limit (UNL)]) and with moderate to markedly elevated (≥1.5 × UNL) liver enzyme levels; (2) NC-HBV (positive for hepatitis B surface antigen); (3) NC-HCV (positive for hepatitis C antibody); and (4) noncirrhotic with HBV/HCV coinfection (NC-HBVHCV).

Information associated with conditions that may increase susceptibility to clinically significant infectious diseases such as diabetes, renal function, BMI, education level, cigarette smoking, alcohol consumption, systemic steroid use, and history of hospitalization were also collected. Diabetes was defined as meeting any of the following criteria: (1) fasting plasma glucose (FPG) over 126 mg/dL or (2) prescription of any antidiabetic agent (verified from the health insurance claims database) for more than 28 days in the previous year before the baseline survey. Participants were classified into the following categories: no diabetes, diabetes with FPG ≤ 130 mg/dL, diabetes with FPG 131–200 mg/dL, and diabetes with FPG > 200 mg/dL. Renal function was defined by estimated glomerular filtration rate (eGFR) using the Chronic Kidney Disease Epidemiology Collaboration (CKD-EPI) study equation. Participants were classified into the following categories according to their eGFR level and whether they received dialysis therapy: ≥90 mL/min/1.73 m^2^, 60–89 mL/min/1.73 m^2^, or <59 mL/min/1.73 m^2^ or receiving dialysis. Information about hemodialysis or peritoneal dialysis was obtained from the NHI database. BMI was calculated by dividing weight (in kilograms) by the square of the patient’s height (in meters). Weight and height were self-reported by the participants. The baseline information on demographic variables (e.g., sex, age, level of education, and marital status) and behavioral risk factors (e.g., smoking and alcohol use) was obtained from the questionnaire completed at cohort entry. Information about comorbid diseases, systemic steroid use for >30 days in the year prior to study entry, and history of hospitalization within 6 months before hospitalization for an infection syndrome were obtained from the NHI database. Inpatient and outpatient diagnosis and prescription files during the 12-month period before study entry were used to ascertain patient comorbidities (including diabetes mellitus, hypertension, cardiovascular disease, cerebrovascular disease, peripheral vascular disease, dyslipidemia, chronic liver and lung disease, autoimmune disease, dementia, cancer, human immunodeficiency virus infection, opioid dependence or abuse, peptic ulcer disease, gastrointestinal bleeding, and proton pump inhibitor/H2-receptor blocker use) and to calculate Charlson comorbidity scores (International Classification of Diseases, ninth revision, Clinical Modification [ICD-9-CM] codes and Anatomical Therapeutic Chemical codes were provided in S1 Table).

#### Outcome and follow-up plan

The primary outcome of interest was incident hospitalization for various prespecified systemic or localized infectious episodes ascertained from the NHI claims database after study initiation. Hospitalizations for infection were further classified according to the specific site of infection—including septicemia; lower respiratory tract, intra-abdominal, reproductive, urinary tract, and skin and soft tissue infections; osteomyelitis; and necrotizing fasciitis—as defined by the ICD-9-CM codes listed in S1 Table. Patients could have more than one specific site of infection during their first hospitalization for infection. We also analyzed infection-related mortality. The vital status and date of death for the study participants was ascertained by linkage with a unique identification number through the National Death Registry. Infection-related death was defined by the death certificate codes according to ICD-9 and ICD-10. The cohort participants were followed from the health screening date up to the first hospitalization for infection, death (based on vital registry), or December 31, 2014, whichever occurred first.

#### Statistical analysis

The number of patients co-infected with HBV and HCV was small; thus, the category of NC-HBVHCV was not included in the analysis. We tabulated the frequency distribution of baseline characteristics and laboratory data, as well as Charlson comorbidity score, among patients in the 4 liver disease groups. Crude incidence rates were calculated for hospitalization for overall and individual sites of infection and infection-related mortality, along with 95% confidence intervals (CIs) using Poisson distribution. The Kaplan–Meier analyses and log-rank tests were used to compare the outcome in different liver disease categories. We used Cox proportional regression models to estimate the hazard ratios (HRs) and 95% CIs of these infections for different liver disease categories and compared these to those of NBNC patients with normal to mildly elevated liver enzyme levels. Important risk factors—including age, sex, BMI, smoking, alcohol consumption, education level, diabetes, eGFR category, systemic steroids use, and history of hospitalization—were chosen and adjusted in the analysis. To avoid overadjustment of intermediate variables on a causal pathway, we did not control for comorbidities (e.g., cardiovascular diseases, autoimmune diseases) that were considered extrahepatic manifestations of HCV infection in the main analysis [27]. We performed sensitivity analyses to investigate the robustness of the results under different assumption-based scenarios and alternative modeling of independent variables, as follows: (1) BMI was adjusted as a categorical variable (following the World Health Organization classification: underweight [<18.5 kg/m^2^], normal weight [≥18.5 kg/m^2^ and <25 kg/m^2^], overweight [≥25 kg/m^2^ and <30 kg/m^2^], and obese [≥30 kg/m^2^]) instead of as a continuous variable using splines in the main analysis, as well as adjusted for continuous FPG and continuous eGFR; (2) we excluded participants with a diagnosis of human immunodeficiency virus infection, opioid dependence or abuse, receiving dialysis, and those with an aspartate aminotransferase to platelet ratio index (APRI) ≥ 1.5; and (3)we excluded patients with NC-HCV or NC-HBV receiving antiviral therapy during the study period, and we additionally controlled for Charlson comorbidity score.

To further explore the mechanism associated with increased susceptibility to infection among the NC-HCV population, we performed stratified analyses in patients with HCV according to the extent of ALT elevation (<1.5 × UNL or ≥1.5 × UNL), APRI (< or ≥ median APRI level) [28], and alcohol use (never or ever alcohol use). We also performed subgroup analyses to investigate whether the risk may be modified by age (≥ 50 versus <50 years of age) and sex. Potential effect modification was assessed by observing overlap of the 95% CIs in the subgroups. All analyses were conducted using SAS software version 9.4 (SAS Institute, Cary, North Carolina).

### Evaluate the impact of anti-HCV treatment on the risks of infectious disease morbidity and mortality

The emerging and promising treatments for HCV, direct-acting antiviral agents (DAAs), were approved in 2014 and covered by NHI since 2017. Because our study period (2005–2014) pre-dated the NHI reimbursement of DAAs, we could not evaluate the effect of DAA treatment on infection risk based on current data. To further address the impact of contemporary viral hepatitis treatment on infection risk, we conducted a retrospective cohort analysis using data from the nationwide NHIRD (23 million beneficiaries).

Patients who had at least one hospital admission or one outpatient visit with HCV or HBV diagnosis between January 1, 2004, and December 31, 2014, were identified from the NHIRD. By using serological test results in the New Taipei City Health Screening Database as the gold standard for HCV and HBV infection, we found a positive predictive value of 87% and 90% and a negative predictive value of 99% and 94% when using the above algorithm to identify patients infected by HCV and HBV, respectively. We excluded patients age 20 years or younger on the dates of first HCV or HBV diagnosis and those with ambiguous sex information. Also excluded were patients who had conditions or factors that substantially increase risk for infections—including those with human immunodeficiency virus infection, organ transplantation, cancer, alcoholism-associated disorders, systematic autoimmune diseases, organ-specific autoimmune diseases, and liver cirrhosis and those who received dialysis. Patients who were censored on the date of first antiviral therapy prescription, those who had both HBV and HCV infection, and prevalent users of antiviral therapy also were excluded.

#### Study drugs and study cohort assembly

We identified hepatitis C–infected patients who initiated ribavirin plus peg-interferon α-2b or ribavirin plus peg-interferon α-2a, the two most common antiviral regimens for HCV treatment, on the same day at outpatient visits between January 1, 2004, and December 31, 2014. For patients with HBV, those who initiated lamivudine, tenofovir, entecavir, or telbivudine at outpatient visits during the same period were identified. Initiation was defined as having been free of any prescription for an antiviral agent 1 year prior to the first prescription date (index date). We defined the date of viral hepatitis diagnosis as the earliest of the following: date of the first outpatient viral hepatitis diagnosis, date of the first admission for viral hepatitis diagnosis, or date of the first antiviral agent dispensing during this window. For each patient with HCV or HBV who never received antiviral therapy during the study period, we randomly assigned a date as the index date, according to the distribution of the index dates of those who initiated antiviral therapy.

#### Follow-up and outcomes

The outcome of interest was the first hospitalization for various prespecified systemic or localized infectious episodes ascertained from the NHI claims database after study initiation. We followed patients from the index date to the earliest of the following: outcome occurrence, death, disenrollment from the NHI program, or December 31, 2015. Patients continued to contribute person-time and number of outcome events regardless of whether their antiviral regimens were changed during the follow-up period.

#### Covariate assessment

Information on potential baseline confounders included age, sex, comorbidities, other medication use, and resource utilization. We assessed comorbidities, other medication use, and resource utilization within 360 days prior to the index date. We determined comorbidities according to inpatient and outpatient diagnosis files and determined other medication use according to pharmacy dispensing claims of outpatient clinics and community pharmacies. We measured resource utilization based on records for hospital admission and outpatient visits.

#### Statistical analyses

Baseline demographics, comorbidities, medication use, and resource utilization among patients with HCV or HBV who received and did not receive antiviral therapy were tabulated and compared with standardized differences, calculated as the difference in means or proportions of a covariate between two groups divided by the pooled standard deviation of the covariate. The crude incidence rates for hospitalization for all infection, specific site of infection, and infection-related death were computed, and their 95% CIs were estimated based on a Poisson distribution.

We estimated baseline propensity scores (PSs) using the covariates associated with demographics, comorbidities, medication use, and resource utilization with a logistic regression model to predict the probability of initiating the study regimen for individual patients. Given that many more patients with HCV or HBV did not receive antiviral therapy than did receive it, we mainly conducted a 1 to 1–5 PS matching (matching each hepatitis patient initiating antiviral therapy to 1–5 patients who never received antiviral therapy) using a nearest-neighbor algorithm with a maximum matching caliper of 0.025 on the PS scale. We assessed whether adequate balance in covariates was achieved using standardized differences, with the values less than 0.1 indicating good balance between comparison groups. We performed Cox proportional hazard modeling to estimate the HR and the 95% CIs for incident hospitalization for infection comparing patients with viral hepatitis who received antiviral therapy to those who did not in the original cohort and in the 1:1–5 PS-matched cohort.

#### Auxiliary analyses

Patients with viral hepatitis who received antiviral therapy differed substantially in liver disease severity from those who did not. The parameters associated with liver disease severity were seldom recorded in the claims database, and these factors may also influence infection risk. For this reason, we undertook additional analyses to mitigate potential residual confounding. We used high-dimensional PS (hd-PS) to identify and include in the PS model an additional 100 empirically identified variables, such as whether the patients underwent evaluation of liver enzymes, HBV/HCV viral load measurement, abdominal sonography, and liver biopsy. The hd-PS is a semiautomated algorithm that identifies, prioritizes, and selects for inclusion in the PS model a large number of covariates that act like confounders based on empirical associations between treatments and outcomes. This approach is accordingly helpful in dealing with covariates that are proxies for confounders but not prespecified or those that are imbalanced between treatments and outcomes because of chance [29].

## Results

### Risks of infectious disease morbidity and mortality among patients with NC-HBV or NC-HCV

**Fig 1:**
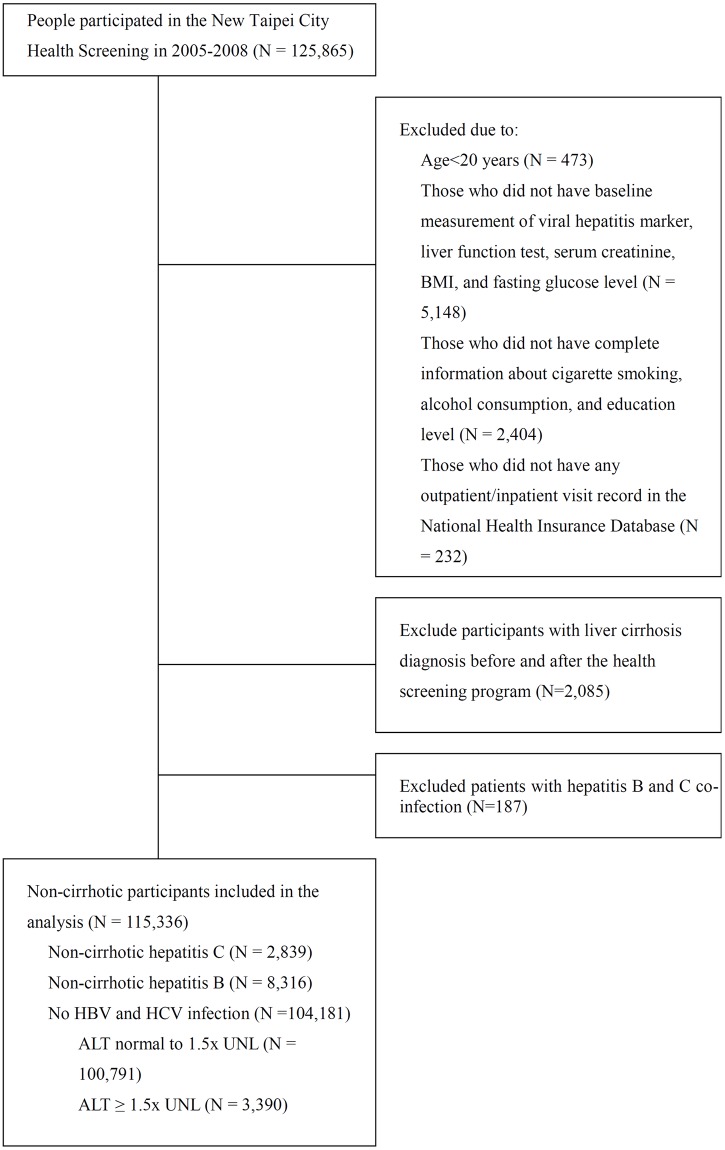
Flow chart for study participants’ enrollment. ALT, alanine aminotransferase; BMI, body mass index; HBV, hepatitis B virus; HCV, hepatitis C virus; UNL, upper normal limit.

A total of 115,336 participants were included in the analysis, with 100,791 NBNC participants with normal to mildly elevated liver enzyme levels, 3,390 NBNC patients with moderate to markedly impaired liver enzyme levels, 8,316 NC-HBV patients, and 2,839 NC-HCV patients (Fig 1). The mean age was 49.7 to 57.0 years among all liver disease categories, and a female predominance pattern was noted in all but the NBNC group with moderate to markedly elevated liver enzyme levels (Table 1). NBNC patients with moderate to markedly elevated liver enzyme levels more often had obesity and consumed more alcohol, and a higher proportion of them had hypertension and poorly controlled diabetes. NC-HCV patients were older, had higher Charlson comorbidity scores and a lower education level, drank less, and had a higher percentage of some comorbidities (including ischemic heart disease, congestive heart failure, peripheral vascular disease, renal failure, dementia, chronic lung disease, stroke, peptic ulcer disease [including those treated with gastric acid–suppressive medications: proton pump inhibitors or H2-receptor blockers]), systemic steroid use, and hospitalization (including because of infection or gastrointestinal bleeding) before entering the study.

**Table 1:** Characteristics of study participants with different liver disease category at study entry (*N* = 115,336).

	NBNC		NC-HBV	NC-HCV
	ALT normal to 1.5 × UNL	ALT ≥ 1.5 × UNL		
***Number***	100,791	3,390	8,316	2,839
Newly diagnosed viral hepatitis*	†	†	75.46	70.66
***Male***	34.60	54.10	39.95	34.52
***Age*, *mean (SD)***	52.36 (11.77)	50.09 (10.74)	49.70 (10.91)	57.04 (11.87)
**20–40**	15.47	20.12	20.61	7.54
**41–50**	31.71	31.68	36.46	23.56
**51–60**	29.27	31.95	26.58	31.21
**61–70**	15.51	12.63	11.77	23.18
**71–100**	8.04	3.63	4.58	14.51
***BMI***				
**Underweight**	2.75	0.50	3.13	3.21
**Normal**	58.92	27.32	58.85	56.78
**Overweight**	31.93	52.01	31.60	32.90
**Obesity**	6.40	20.18	6.42	7.12
***Cigarette smoking***				
**Never**	79.33	69.03	77.02	76.40
**Quitted**	6.45	10.06	6.71	6.31
**Current**	14.22	20.91	16.27	17.29
***Alcohol consumption***				
**Never**	60.78	52.48	60.03	68.47
**Quitted**	1.92	2.89	1.97	3.59
**Regular**	6.93	10.74	7.46	6.55
**Sometimes**	30.37	33.89	30.54	21.38
***Education level***				
**Illiterate**	7.79	6.58	6.66	17.29
**Literate but not attending elementary school**	2.12	1.80	1.62	4.12
**Elementary school**	25.46	22.95	22.44	33.96
**Junior high school**	16.24	16.17	17.85	16.63
**High school**	28.19	29.09	30.48	18.63
**College**	18.53	21.00	19.02	8.70
**Graduate school**	1.68	2.42	1.92	0.67
***Systemic steroid use >30 days before study beginning***	1.30	1.45	0.96	2.32
***History of hospitalization within 6 months before study entry***	2.75	3.48	2.55	4.54
***History of hospitalization within 6 months before hospitalization for infection syndrome***	2.42	2.51	1.97	5.11
***History of hospitalization for infection within 6 months before study entry***	0.43	0.53	0.32	0.70
***Comorbidities (%)***				
**Diabetes**				
Fasting glucose ≤90	0.18	0.21	0.14	0.35
Fasting glucose 91–130	2.13	3.57	1.54	2.85
Fasting glucose 131–200	4.61	11.92	3.56	6.34
Fasting glucose >200	1.73	4.37	1.78	2.61
**Hypertension**	18.71	25.37	14.65	23.99
**Ischemic heart disease**	6.05	6.58	4.50	8.03
**Myocardial infarction**	0.21	0.24	0.30	0.39
**Cardiac dysrhythmia/atrial fibrillation**	2.75	2.83	2.20	3.52
**Congestive heart failure**	1.31	1.30	1.00	2.78
**Stroke**	1.38	1.50	0.89	2.57
**Peripheral vascular disease**	0.44	0.35	0.34	0.81
**Disorders of lipid metabolism**	11.87	17.37	9.11	13.70
**Chronic lung disease**	7.61	7.64	6.73	12.89
**eGFR**				
≥90	56.14	57.40	59.13	45.40
60–89	38.22	38.47	37.27	45.23
<59 or on dialysis therapy	5.64	4.13	3.61	9.37
**Autoimmune disease**	2.72	2.98	2.18	3.10
**Dementia**	0.23	0.15	0.06	0.63
**Cancer**	1.88	1.62	1.64	2.43
**Human immunodeficiency virus infection**	<0.01	0.00	0.00	0.00
***Opioid dependence or abuse***	<0.01‡			
***Charlson comorbidity score***	0.41 (0.86)	0.52 (0.94)	0.47 (0.84)	0.77 (1.12)
***Prior history of peptic ulcer disease within 1 year before screening program***	18.11	17.52	17.71	25.36
***Proton pump inhibitor or H2-receptor blocker use within 1 year before screening program***	16.19	16.02	16.70	21.63
***Hospitalization for gastrointestinal bleeding within 1 year before screening program***	0.31	0.21	0.28	0.95
***Laboratory data (mean*, *SD)***				
**Albumin (g/dL)**	4.60 (0.26)	4.70 (0.28)	4.62 (0.26)	4.52 (0.28)
**AST (U/L)**	23.40 (6.40)	58.59 (37.24)	27.88 (19.40)	38.31 (32.59)
**ALT (U/L)**	22.30 (9.36)	89.57 (48.49)	30.34 (29.97)	42.81 (49.58)
**APRI**	0.22 (0.11)	0.58 (0.48)	0.29 (0.27)	0.44 (0.50)
**APRI category (%)**§				
< 0.5	98.64	57.54	93.34	78.00
≥0.5, <1	1.24	33.72	5.46	15.34
≥1, <1.5	0.07	5.61	0.52	3.24
≥1.5, <2	0.02	1.62	0.39	1.66
≥2	0.02	1.51	0.29	1.76

*Newly diagnosed viral hepatitis was defined as no records of diagnostic codes regarding viral hepatitis in administrative database within 1 year before participation in the screening program.

^†^Not applicable.

^‡^The exact case numbers in either category were too small to be retrieved because of the authority’s policy regulation.

^§^The number of participants who had available APRI data were 100,710 for NBNC with ALT normal to 1.5× UNL, 3,387 for NBNC with ALT ≥ 1.5× UNL, 8,309 for NC-HBV, and 2,836 for NC-HCV.

**Abbreviations**: ALT, alanine aminotransferase; APRI, AST to Platelet Ratio Index; AST, aspartate transaminase; BMI, body mass index; eGFR, estimated glomerular filtration rate; HBV, hepatitis B virus; HCV, hepatitis C virus; NBNC, no HBV or HCV infection; NC-HBV, noncirrhotic with HBV infection; NC-HCV, noncirrhotic with HCV infection; UNL, upper normal limit

The characteristics of participants who were included in the final analysis, those who were excluded because of missing data, and the random sample of 1 million nationwide NHI beneficiaries in 2010 are summarized in S2 Table. We found that participants excluded in the final analysis were younger and thus had a lower percentage of diabetes, hypertension, ischemic heart disease, and disorders of lipid metabolism. Compared with the random sample of 1 million nationwide NHI beneficiaries in 2010, the study participants ultimately included had a higher percentage of women and those ages 41 to 70 years, although similar distributions of comorbidities were noted between included study participants and the general population.

During a median follow-up of 8.2 years, 70.9% of those categorized as NC-HCV had been prescribed gastric acid–suppressive medications, and 7.19% had been hospitalized for gastrointestinal bleeding. Similarly, 75.90% and 9.09% of patients in the NC-HBVHCV category received acid-suppressive agents and were admitted for gastrointestinal bleeding, respectively. In contrast, the prevalences in other categories were 61.2%–63.2% and 3.48%–3.85% in NBNC and NC-HBV participants, respectively. Meanwhile, 339 (12.10%) of NC-HCV and 339 (4.09%) of NC-HBV patients received antiviral therapy for viral hepatitis during the study period.

**Fig 2:**
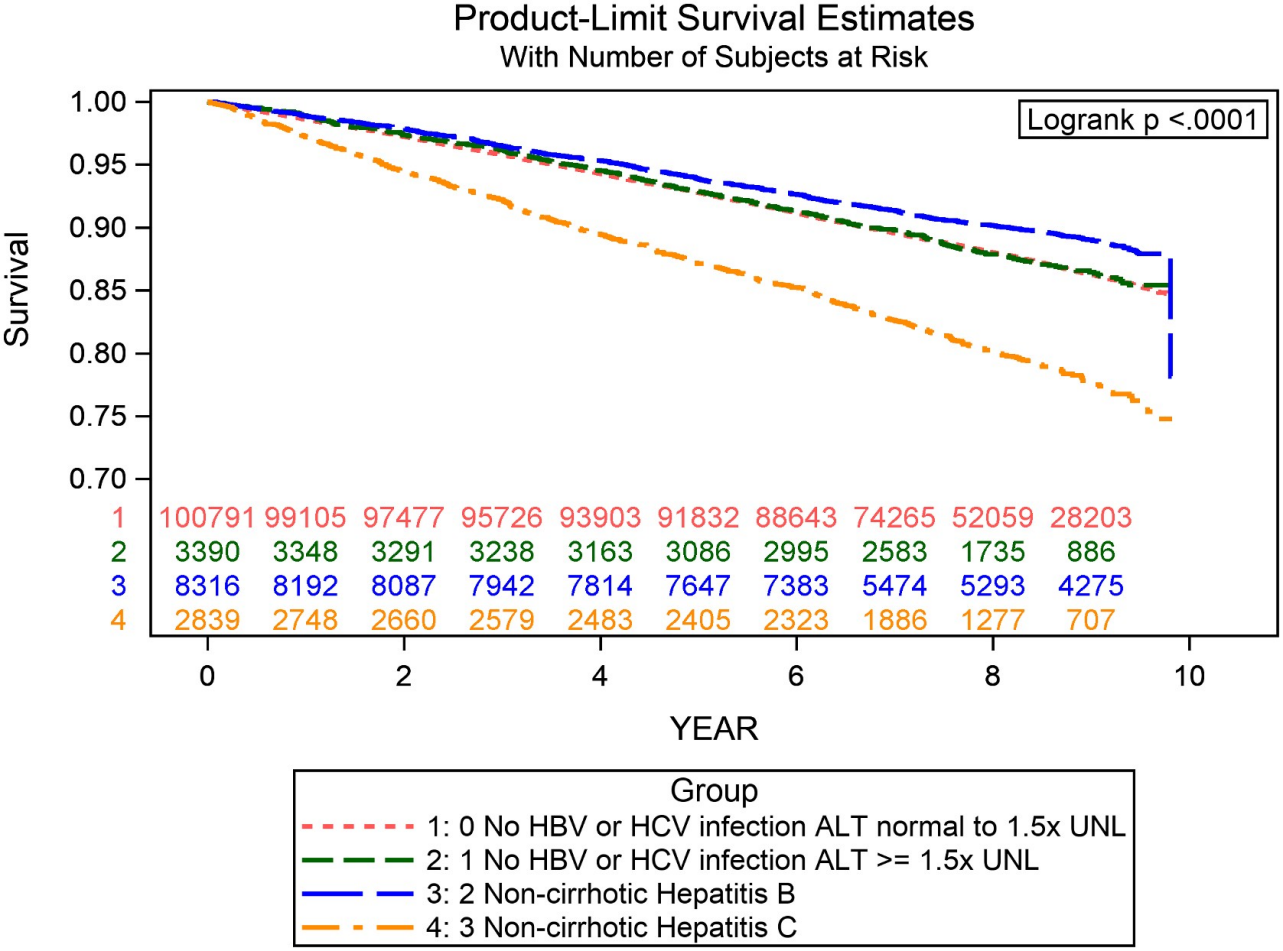
The Kaplan-Myer curves for the incidence of any hospitalization due to infection in different liver disease categories. The red line (“no HBV or HCV infection ALP normal to 1.5xUNL”) represents NBNC participants with normal to mildly elevated liver enzyme; the green line (“no HBV or HCV infection ALP ≥ 1.5x UNL”) represents NBNC participants with moderate to markedly elevated liver enzyme; the blue line (“non-cirrhotic Hepatitis B”) represents patients with NC-HBV; and the orange line (“non-cirrhotic Hepatitis C”) represents patients with NC-HCV. ALT, alanine aminotransferase; HBV, hepatitis B virus; HCV, hepatitis C virus; UNL, upper normal limit.

**Fig 3:**
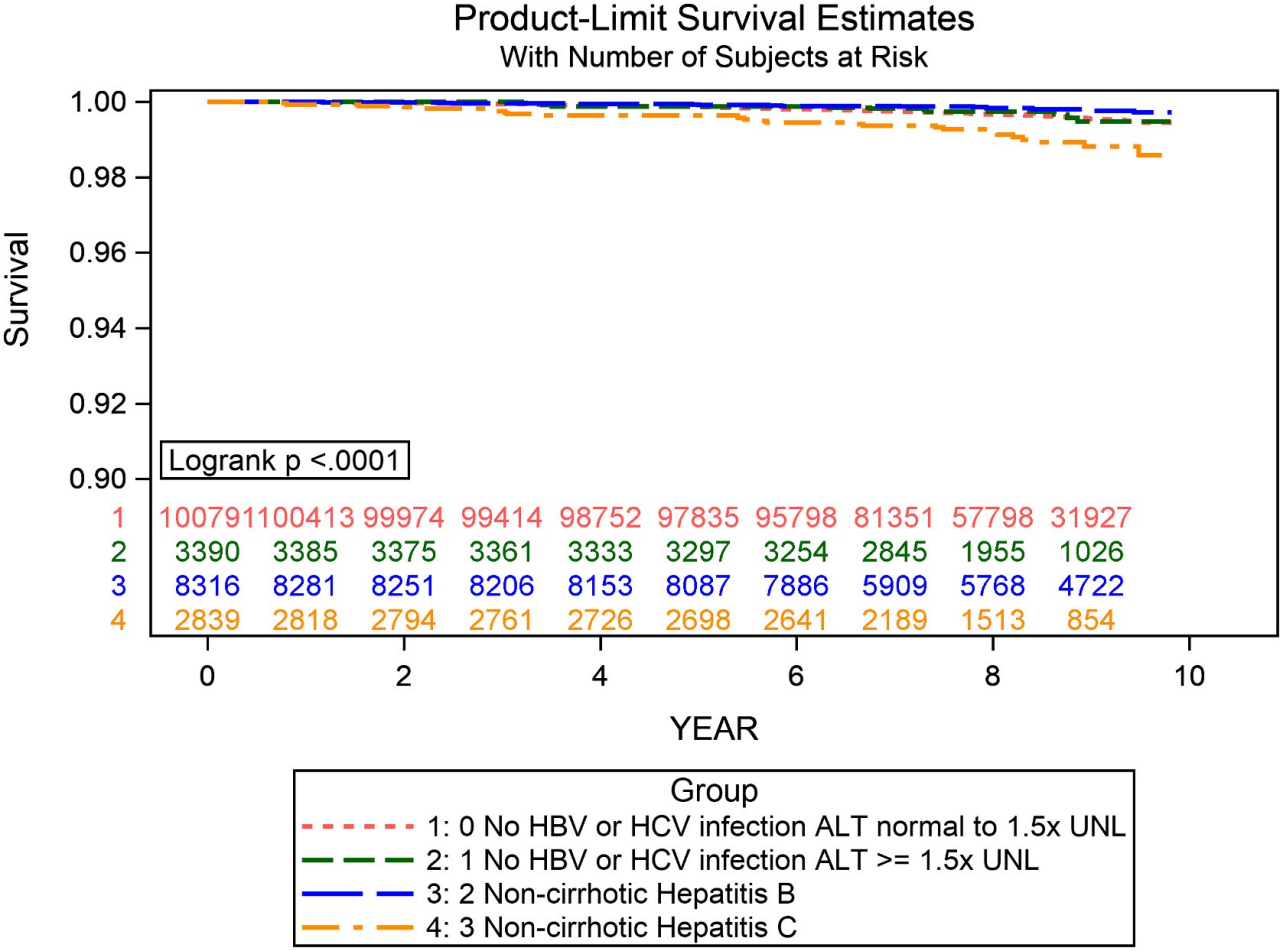
The Kaplan-Myer curves for infection-related mortality in different liver disease categories. The red line (“no HBV or HCV infection ALP normal to 1.5x UNL”) represents NBNC participants with normal to mildly elevated liver enzyme; the green line (“no HBV or HCV infection ALP ≥ 1.5x UNL”) represents NBNC participants with moderate to markedly elevated liver enzyme; the blue line (“non-cirrhotic Hepatitis B”) represents patients with NC-HBV; and the orange line (“non-cirrhotic Hepatitis C”) represents patients with NC-HCV. ALT, alanine aminotransferase; HBV, hepatitis B virus; HCV, hepatitis C virus; UNL, upper normal limit.

During follow-up, a total of 14,279 incident cases of infection leading to hospitalization were identified (Table 2). The crude incidence rate for overall infection was 16.13 (95% CI: 15.84–16.41) and 16.04 (95% CI: 14.57–17.66) per 1,000 person-years in NBNC patients with normal to mildly elevated and moderate to markedly elevated liver enzymes, respectively; 13.11 (95% CI: 12.27–14.01) per 1,000 person-years in NC-HBV patients; and 27.94 (95% CI: 25.74–30.32) per 1,000 person-years in NC-HCV patients. Patients infected with HCV had the highest crude incidence rate for all infections, certain site-specific infections (especially septicemia, lower respiratory tract, and reproductive and urinary tract infection), and infection-related deaths. The event-free probabilities for overall infection and infection-related mortality were lower in the NC-HCV category than in other liver disease categories (Figs 2 and 3, log-rank *p* < 0.0001).

**Table 2:** Follow-up duration, number of incident cases, and crude incidence of hospitalization for infection syndrome and infection-related mortality (*N* = 115,336).

	NBNC		NC-HBV	NC-HCV
	ALT normal to 1.5 × UNL	ALT ≥ 1.5 × UNL		
***N***	100,791	3,390	8,316	2,839
***Follow-up duration***				
**Total person-years**	770,446	25,929	66,122	20,476
**Median follow-up years (interquartile range)**	8.18 (2.39)	8.15 (1.92)	9.05 (2.92)	7.64 (2.50)
***Hospitalization for infection***				
**All infections**				
Number of incidence cases	12,424	416	867	572
Crude incidence rate^†^	16.13 (15.84–16.41)	16.04 (14.57–17.66)	13.11 (12.27–14.01)	27.94 (25.74–30.32)
**Septicemia**				
Number of incidence cases	2,180	64	135	112
Crude incidence rate^†^	2.83 (2.71–2.95)	2.47 (1.93–3.15)	2.04 (1.72–2.42)	5.47 (4.55–6.58)
**Lower respiratory tract**				
Number of incidence cases	3,508	93	208	182
Crude incidence rate^†^	4.55 (4.40–4.71)	3.59 (2.93–4.40)	3.15 (2.75–3.60)	8.89 (7.69–10.28)
**Intra-abdominal**				
Number of incidence cases	1,619	77	110	63
Crude incidence rate^†^	2.10 (2.00–2.21)	2.97 (2.38–3.71)	1.66 (1.38–2.01)	3.08 (2.40–3.94)
**Reproductive and urinary tract**				
Number of incidence cases	4,949	151	373	235
Crude incidence rate^†^	6.42 (6.25–6.61)	5.82 (4.97–6.83)	5.64 (5.10–6.24)	11.48 (10.10–13.04)
**Skin and soft tissue**				
Number of incidence cases	1,594	57	116	55
Crude incidence rate^†^	2.07 (1.97–2.17)	2.20 (1.70–2.85)	1.75 (1.46–2.10)	2.69 (2.06–3.50)
**Osteomyelitis**				
Number of incidence cases	228	8	11	9
Crude incidence rate^†^	0.3 (0.26–0.34)	0.31 (0.15–0.62)	0.17 (0.09–0.30)	0.41 (0.21–0.79)
**Necrotizing fasciitis**				
Number of incidence cases	46	NA*	NA*	3
Crude incidence rate^†^	0.06 (0.04–0.08)	NA*	NA*	0.15 (0.05–0.45)
**Infectious intestinal diseases**				
Number of incidence cases	333	10	22	16
Crude incidence rate^†^	0.43 (0.39–0.48)	0.39 (0.21–0.72)	0.33 (0.22–0.51)	0.73 (0.45–1.19)
**Infection-related deaths**				
Number of cases	369	11	17	26
Crude mortality rate^†^	0.45 (0.41–0.50)	0.40 (0.22–0.72)	0.25 (0.15–0.39)	1.27 (0.86~1.86)

*Total case number of necrotizing fasciitis among participants without viral hepatitis but with moderate-to-marked impaired liver function and those with NC-HBV was 4. The exact case number in each category could not be retrieved because of the authority’s policy regulation.

^†^Crude incidence rate or mortality rate per 1,000 person-years.

**Abbreviations**: ALT, alanine aminotransferase; HBV, hepatitis B virus; HCV, hepatitis C virus; NA, not applicable; NBNC, no HBV or HCV infection; NC-HBV, noncirrhotic with HBV infection; NC-HCV, noncirrhotic with HCV infection; UNL, upper normal limit

Table 3 presents results from the Cox proportional hazard regression analysis that used NBNC participants with normal to mildly elevated liver enzyme levels as a reference group. After controlling for important risk factors, NC-HCV patients had a significantly increased risk for overall infection (adjusted HR: 1.22; 95% CI: 1.12–1.33) but not for infection-related mortality (adjusted HR: 1.37; 95% CI: 0.92–2.05). Significantly increased risks were also found for septicemia and lower respiratory tract, reproductive, and urinary tract infections. In contrast, NBNC patients with moderate to markedly elevated liver enzyme levels and NC-HBV patients were not associated with overall infections. The adjusted HR for hospitalization for overall infection was 0.94 (95% CI: 0.88–1.01) for the NC-HBV group and 1.03 (95% CI: 0.93–1.14) for NBNC with moderate to markedly elevated ALT levels. Being in these two groups was not associated with a higher risk for any site-specific infection or infection-related death. Similar results were found in the sensitivity analysis that adjusted for categorical BMI and continuous FPG and eGFR; excluded high-risk populations, such as those with human immunodeficiency virus infection, opioid dependence or abuse, dialysis, or high APRI; excluded patients receiving treatment during follow-up; and additionally controlled for Charlson comorbidity score (S4–S6 Tables).

**Table 3:** The association between different liver disease categories and risk of hospitalization for infection syndrome and infection-related mortality compared with NBNC patients with normal to mildly elevated liver enzyme levels (*N* = 115,336).

Hospitalization for infection	NBNC			NC-HBV		NC-HCV	
	ALT normal to 1.5 × UNL	ALT ≥ 1.5 × UNL					
	HR	Crude HR	Adjusted HR*	Crude HR	Adjusted HR*	Crude HR	Adjusted HR*
**All infections**	1.0 (Reference)	1.00 (0.90–1.10)	1.03 (0.93–1.14)	0.80 (0.75–0.86)	0.94 (0.88–1.01)	1.74 (1.60–1.89)	1.22 (1.12–1.33)
**Septicemia**	1.0 (Reference)	0.87 (0.68–1.12)	0.96 (0.75–1.23)	0.71 (0.59–0.84)	0.89 (0.75–1.06)	1.95 (1.61–2.36)	1.26 (1.04–1.53)
**Lower respiratory tract**	1.0 (Reference)	0.79 (0.64–0.97)	1.02 (0.83–1.25)	0.68 (0.59–0.78)	0.90 (0.78–1.04)	1.96 (1.69–2.28)	1.25 (1.08–1.45)
**Intra-abdominal**	1.0 (Reference)	1.41 (1.13–1.78)	1.25 (0.99–1.58)	0.79 (0.65–0.95)	0.81 (0.67–0.99)	1.47 (1.14–1.89)	1.22 (0.95–1.58)
**Reproductive and urinary tract**	1.0 (Reference)	0.91 (0.77–1.07)	0.95 (0.81–1.12)	0.87 (0.78–0.96)	1.00 (0.90–1.11)	1.79 (1.57–2.04)	1.28 (1.12–1.46)
**Skin and soft tissue**	1.0 (Reference)	1.06 (0.82–1.38)	0.85 (0.65–1.12)	0.85 (0.70–1.02)	0.97 (0.80–1.17)	1.30 (0.99–1.70)	0.93 (0.71–1.22)
**Osteomyelitis**	1.0 (Reference)	1.05 (0.52–2.12)	1.01 (0.50–2.07)	0.55 (0.30–1.01)	0.66 (0.36–1.20)	1.48 (0.76–2.88)	0.94 (0.48–1.84)
**Necrotizing fasciitis**	1.0 (Reference)	0.65 (0.09–4.69)	0.43 (0.06–3.16)	0.76 (0.24–2.44)	0.86 (0.27–2.77)	2.44 (0.76–7.86)	1.57 (0.48–5.11)
**Infectious intestinal diseases**	1.0 (Reference)	0.89 (0.48–1.68)	0.91 (0.48–1.71)	0.76 (0.49–1.17)	0.83 (0.54–1.28)	1.81 (1.10–2.99)	1.37 (0.83–2.28)
**Infection-related deaths**	1.0 (Reference)	0.89 (0.49–1.62)	1.52 (0.83–2.79)	0.50 (0.31–0.82)	0.87 (0.53–1.41)	2.58 (1.73–3.84)	1.37 (0.92–2.05)

*Adjusted for continuous age, sex, BMI (spline function with 3 knots), smoking (current, noncurrent), alcohol consumption, education level, FPG (categorical), eGFR (categorical), systemic steroids use >30 days before study entry, and history of hospitalization within 6 months before hospitalization for infection syndrome.

**Abbreviations**: ALT, alanine aminotransferase; BMI, body mass index; eGFR, estimated glomerular filtration rate; FPG, fasting plasma glucose; HBV, hepatitis B virus; HCV, hepatitis C virus; HR, hazard ratio; NBNC, no HBV or HCV infection; NC-HBV, noncirrhotic with HBV infection; NC-HCV, noncirrhotic with HCV infection; UNL, upper normal limit

In the analyses exploring possible factors associated with increased susceptibility to infection in NC-HCV patients, consistently elevated risks for hospitalizations from all infections were noted regardless of ALT level and APRI (S7 Table). With regard to alcohol use, those who reported never drinking alcohol still had a significantly increased risk for overall infection. Otherwise, patients with low APRI had a higher risk for infection-related mortality (adjusted HR: 2.23; 95% CI: 1.35–3.69). Because of small patient numbers after stratification, no precise estimation was possible, especially for the risk of site-specific infections.

In the subgroup analysis, the association between NC-HCV and overall infection was statistically significant in patients aged over 50 years (S8 and S9 Tables). Otherwise, no obvious effect modification by sex was noted in patients with HCV with regard to overall hospitalized infection and infection-related mortality (S10 and S11 Tables).

### Evaluate the impact of anti-HCV treatment on the risks for infectious diseases morbidity and mortality

#### The impact of HCV antiviral treatment on infection risks

**Fig 4:**
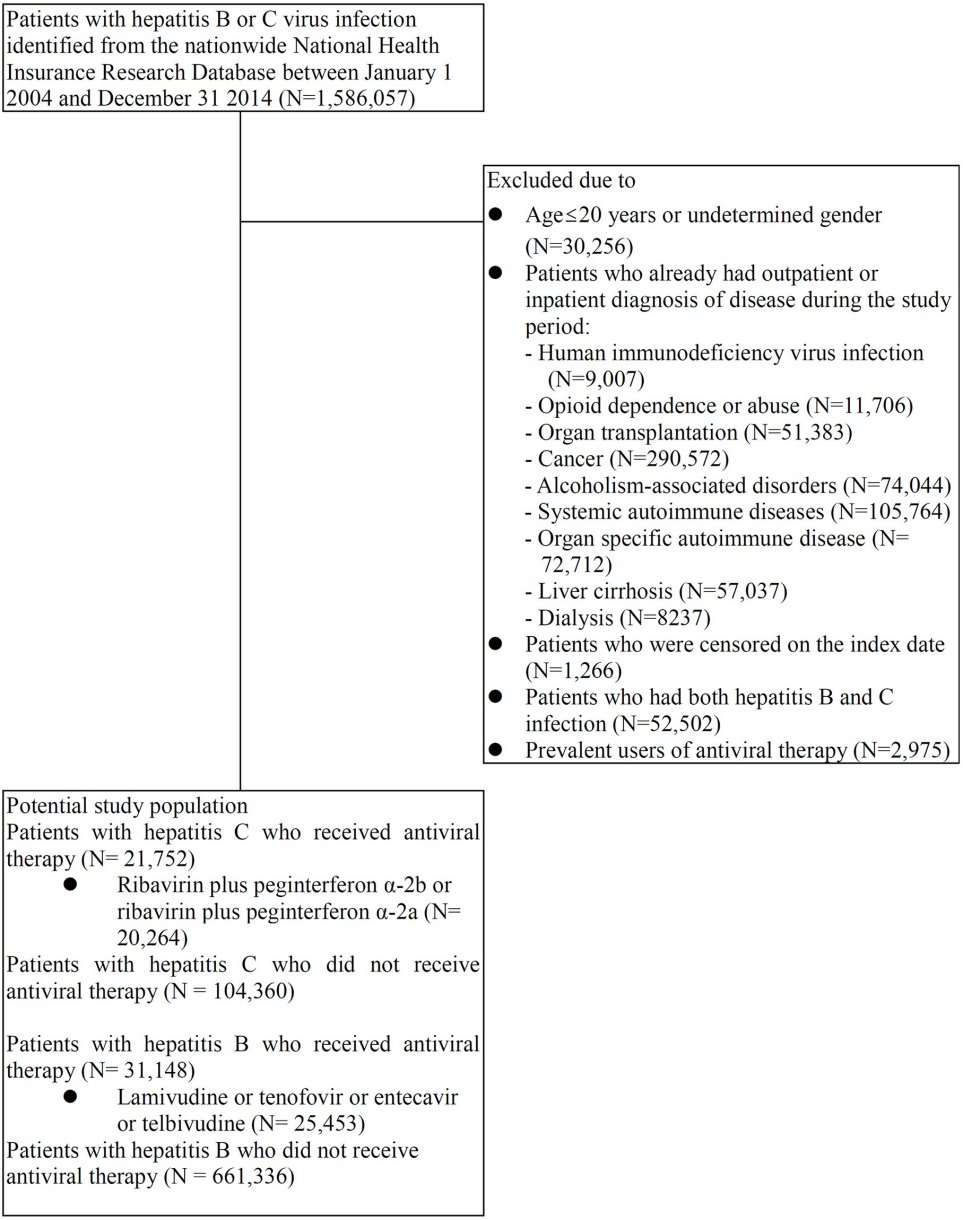
Assembly of study cohorts that evaluate the impact of antiviral treatment on the risks for infectious diseases morbidity and mortality in HCV and HBV patients.

We identified 124,624 eligible patients with HCV (20,264 initiated the study regimen of ribavirin plus peg-interferon α-2b or ribavirin plus peg-interferon α-2a and 104,360 patients did not receive antiviral therapy) (Fig 4).

Before PS matching, patients with chronic HCV initiating antiviral therapy were younger, more likely to be men, and more likely to have moderate–severe liver disease and peptic ulcer disease, but they were less likely to have comorbidities such as congestive heart failure, stroke, chronic lung disease, and dementia and thus had a lower Charlson comorbidity score. Meanwhile, patients receiving treatment for HCV were less likely to have experienced hospitalization because of infection-related episodes but more likely to have had hospitalizations because of liver disease–related episodes compared to patients who did not receive antiviral therapy (S12 Table).

To further explore the potential differences in baseline characteristics between patients with treated versus untreated HCV, we also analyzed data from the New Taipei City Health Screening Program. Patients with HCV who received antiviral therapy were more likely to have a higher education level and higher eGFR, although they also had higher liver enzymes and APRI scores compared with patients with untreated HCV (S3 Table).

In the PS-matching analysis that included 68,723 HCV-infected patients (16,558 initiated with antiviral therapy and 52,165 untreated patients), improvement of balance in baseline characteristics was observed between two comparison groups.

The median follow-up time was 4.2 years for HCV-infected patients who received antiviral therapy and 4.1 years for patients with untreated HCV (S13 Table). The crude incidence rate of hospitalization for all infections was substantially lower among patients with HCV who received antiviral therapy (14.87; 95% CI: 14.09–15.69 per 1,000 person-years) compared with those who did not receive antiviral therapy (25.99; 95% CI: 25.52–26.47 per 1,000 person-years). Lower crude incidence rates were also noted among treated patients with HCV and certain site-specific infections (septicemia, lower respiratory tract, reproductive/urinary tract and skin and soft tissue infection). After PS matching, the adjusted incidence rates of overall infection and some site-specific infection—including septicemia and lower respiratory tract and reproductive/urinary tract infection—remained lower in the treated group compared with the untreated group. Patients with treated HCV also had lower infection-related mortality rates compared with the untreated group both before (0.13 [95% CI: 0.07–0.22] versus 3.31 [95% CI: 3.15–3.48] per 1,000 person-years) and after (0.13 [95% CI: 0.05–0.33 versus 1.32 [95% CI: 1.18–1.47] per 1,000 person-years) PS matching.

Table 4 presents results from the Cox proportional hazard regression analysis in which HCV-infected patients who did not receive antiviral therapy were considered a reference group. After PS matching, among patients with HCV, having received antiviral therapy was associated with a significantly reduced risk for hospitalization for all infections (HR: 0.79; 95% CI: 0.73–0.84) and infection-related deaths (HR: 0.08; 95% CI: 0.04–0.16). Lower risks were also found for many site-specific infections—including septicemia; lower respiratory tract, reproductive, urinary tract, and skin and soft tissue infections; and necrotizing fasciitis. As noted, we also performed an analysis aimed at reducing residual confounding by hd-PS, with better comparability in terms of baseline characteristics among comparison groups. In this analysis, although risk estimates shifted more toward the null, a consistent reduction in risks of hospitalization for all infections (adjusted HR: 0.88; 95% CI: 0.82–0.94) and infection-related deaths (adjusted HR: 0.13; 95% CI: 0.07–0.25) was still found in patients with HCV treated by antiviral therapy compared with untreated patients.

**Table 4:** Risk of hospitalization for infection syndrome and infection-related mortality comparing HCV patients who received antiviral therapy with those who did not receive antiviral therapy.

Hospitalization for infection	HCV patients who did not receive antiviral therapy	HCV patients who received antiviral therapy		
	HR	HR before PS matching (95% CI)	HR after PS matching (95% CI)	HR after hd-PS matching (95% CI)
**All infections**	Reference	0.57 (0.54–0.60)	0.79 (0.73–0.84)	0.88 (0.82–0.94)
**Septicemia**	Reference	0.45 (0.40–0.51)	0.71 (0.61–0.82)	0.86 (0.74–1.01)
**Lower respiratory tract**	Reference	0.37 (0.34–0.42)	0.58 (0.51–0.66)	0.69 (0.60–0.78)
**Intra-abdominal**	Reference	0.88 (0.75–1.03)	0.97 (0.80–1.18)	0.97 (0.79–1.18)
**Reproductive and urinary tract**	Reference	0.50 (0.46–0.56)	0.82 (0.74–0.92)	0.93 (0.83–1.04)
**Skin and soft tissue**	Reference	0.73 (0.63–0.84)	0.80 (0.68–0.95)	0.91 (0.77–1.09)
**Osteomyelitis**	Reference	0.88 (0.61–1.29)	1.15 (0.75–1.78)	1.28 (0.82–2.01)
**Necrotizing fasciitis**	Reference	0.49 (0.27–0.89)	0.47 (0.23–0.93)	0.41 (0.17–0.98)
**Infectious intestinal diseases**	Reference	1.06 (0.83–1.37)	1.13 (0.84–1.54)	1.24 (0.91–1.70)
**Infection-related deaths**	Reference	0.04 (0.02–0.07)	0.08 (0.04–0.16)	0.13 (0.07–0.25)

**Abbreviations**: CI, confidence interval; HCV, hepatitis C virus; hd-PS, high-dimensional propensity score; HR, hazard ratio; PS, propensity score

#### The impact of HBV antiviral treatment on infection risks

A total of 686,789 eligible patients with HBV (25,453 who initiated the study regimen of lamivudine, tenofovir, entecavir, or telbivudine and 661,336 who did not receive antiviral therapy) were included in the analysis (Fig 4). Patients receiving anti-HBV treatment were younger and predominantly male, were more likely to have moderate–severe liver and peptic ulcer disease, and had a slightly higher Charlson comorbidity score compared with patients whose HBV was untreated (S14 Table). They were also more likely to have experienced any cause of hospitalization or hospitalization because of infection, liver disease, and gastrointestinal bleeding compared to patients who did not receive antiviral therapy. Similar to patients treated for HCV, in the analysis of data from the New Taipei City Health Screening Program, patients with treated HBV were more likely to have a higher education level, although they also had higher liver enzyme values and APRI scores compared with untreated patients (S3 Table). A better balance of baseline characteristics between treated and untreated groups was reached after matching with PS and hd-PS.

With a median follow-up of 4.4 years, the crude incidence of overall hospitalization for infection was 8.71 (95% CI: 8.20–9.25) per 1,000 person-years for patients with HBV who received antiviral therapy and 9.09 (95% CI: 8.98–9.19) per 1,000 person-years for untreated patients, lower than that for the HCV-infected population (S15 Table). A higher crude incidence for reproductive/urinary tract and skin/soft tissue infection was observed among patients with HBV who did not receive antiviral therapy. The incidence rates of overall infection after matching for PS and hd-PS were 8.56 (95% CI: 8.04–9.63) and 8.62 (95% CI: 8.09–9.18) per 1,000 person-years, respectively, for patients with HBV who received antiviral therapy and 10.48 (95% CI: 9.93–11.06) and 10.08 (95% CI: 9.54–10.66) per 1,000 person-years for untreated patients. A higher incidence rate for skin and soft tissue infection was still noted among patients who did not receive antiviral therapy after PS matching.

Compared with untreated patients with HBV, patients treated for HBV infection had reduced risks for hospitalization for all infections after matching for PS (adjusted HR: 0.80; 95% CI: 0.73–0.87) and hd-PS (adjusted HR: 0.83; 95% CI: 0.76–0.90) (S16 Table). With regard to site-specific infections, a decrease in risk for lower respiratory tract (adjusted HR: 0.80; 95% CI: 0.73–0.87) and skin and soft tissue infection (adjusted HR: 0.53; 95% CI: 0.43–0.65) was observed in patients with HBV receiving antiviral therapy after matching for PS. Similar results were found in the analysis matching for hd-PS. A lower risk for infection-related death was also observed by hd-PS analysis in patients treated for HBV.

## Discussion

In this community-based cohort study of volunteers participating in a health screening program, the presence of a NC-HCV infection conferred a greater risk for hospitalization for overall infections, including sepsis and different infection syndromes, particularly among those over age 50. In contrast, no increased risk for infection-related hospitalization was observed in the NC-HBV and NBNC groups with moderate to markedly elevated ALT levels, most of whom had alcoholic or fatty liver disease.

Because of the mechanism of immune dysfunction (portosystemic shunting, defective cellular defense, and low complement levels), along with excessive activation of pro-inflammatory cytokines, patients with liver cirrhosis are immunocompromised and susceptible to infections [5,6]. We also observed a higher risk for infection in patients in the NC-HCV category. As shown in our exploratory stratified analysis, the elevated risk was independent of ALT level or APRI, which serve as markers of liver injury and fibrosis, respectively. These results suggest a possible unique role for HCV in causing immune dysfunction. Studies have uncovered several alterations associated with peripheral immune depression in patients with HCV infection, including depressed phagocytosis and the killing capacity of polymorphonuclear cells and monocytes; reduced CD3+, CD8+, and CD16+ cell counts; impaired CD4+CD8+-dependent antibacterial activity; and dendritic cell decrease/dysfunction [8–10]. Dolganiuc and colleagues found a unique pattern of Toll-like receptor (TLR) expression in monocytes and T lymphocytes, by which pathogens are recognized to shape the innate and adaptive immune responses, along with elevated TLR RNA expression, which may contribute to susceptibility to infections in patients infected with HCV [11]. Inhibition of the complement system and functional impairment of the membrane attack complex were also noted in patients infected with HCV [12]. Other factors attributed to increased infection risks include receiving interferon-based therapy for viral hepatitis that commonly causes the adverse events of neutropenia and clinically significant infections [30–32]. In our study, only 6% of patients in the NC-HBV and NC-HCV categories received antivirals—including interferon-based therapy—during the study period. However, after excluding those receiving antiviral therapy during follow-up, a consistently increased risk for overall infection was observed among patients with HCV (S6 Table). Additionally, gut dysbiosis and increased bacterial translocation may play a role in explaining the increased risk of infection in patients infected with HCV [33,34]. One study demonstrated a higher change in gut microbiota in cirrhotic patients with HCV compared with those with cirrhosis and HBV [35]. Finally, although the pattern of HCV transmission in Taiwan is mainly iatrogenic and less commonly associated with high-risk behaviors (e.g., illicit drug use, high-risk sexual behavior), shared risk factors such as low socioeconomic status, poverty, or frequent exposure to medical injection or acupuncture may also predispose these patients to clinically generalized or localized infections.

Few clinical research studies have extensively evaluated the association between HCV infection and the risk of sepsis and specific infection syndromes. Only one study examined the risk of several predefined infections among HCV-infected patients. In a large, hospital-based case-control study based on veterans, El-Serag and colleagues reported that patients with HCV, with or without cirrhosis, had a significantly higher prevalence of other blood-borne viral infections, immunodeficiency-related infections, tuberculosis, sexually transmitted diseases, and bacterial infections than patients without HCV infections [7]. Although the sample was predominantly male and possibly had different patterns of HCV transmission [36], the study presented the same argument: that HCV infection may be associated with certain infections, including bacterial, viral, or fungal infections. Other studies have demonstrated that HCV infection is a prognostic or risk factor for some pathogen-specific infections. Among patients hospitalized with invasive pneumococcal disease, Marrie and colleagues found that the existence of anti-HCV antibodies was associated with a higher risk of in-hospital mortality and complications [13]. In a population-based surveillance study in the Calgary Health Region, Canada, patients with HCV had a 5-fold increased relative risk for *Staphylococcus aureus* bacteremia [15]. The presence of HCV seropositivity is a risk factor for bloodstream or bacterial infections following certain procedures, including hemodialysis via tunneled vascular access catheters [18], kidney transplantation [21], transjugular intrahepatic portosystemic shunting [20], and allogeneic hematopoietic cell transplantation [19]. In addition, HCV is suspected as a risk factor or a common coexisting condition for skin/soft tissue infection and necrotizing fasciitis [16,17,23]. Our study showed a nonsignificant increase in the risk for necrotizing fasciitis.

Although more cases are needed for a precise estimate of some site-specific or localized infections, our research suggested that in addition to bloodstream infections, HCV-infected patients might also be prone to certain individual sites of infections such as lower respiratory and reproductive/urinary tract infections, and probably necrotizing fasciitis. Thus, as suggested in guidelines of infection prevention for patients with cirrhosis, avoidance of raw or uncooked foods—especially seafood—and close contact with at-risk animals or sick people and wound exposure to flood or seawater may be applicable to patients with HCV [6]. Healthcare professionals should encourage HCV-infected patients to employ preventive strategies to reduce the risk for infection and to take special precautions when encountering patients with HCV who have initial symptoms of infection. Screening for HCV might be considered if patients exhibit the clinical phenomena of immune dysfunction such as recurrent episodes of systemic or site-specific infections. Whether vaccination against the influenza virus and invasive pneumococcal disease will attenuate infection-related mortality and morbidity among patients with HCV needs to be rigorously evaluated in the future.

Little is known about the risk of infection among patients with nonalcoholic fatty liver disease (NAFLD). However, some studies have suggested a higher risk of bacterial infection among these patients through the mechanisms of diabetes and obesity, which are shared risk factors with NAFLD and, probably, altered immunity [37,38]. Changes in gut microbiota, bacterial overgrowth, increased gut permeability, and increased lipopolysaccharide levels have also been described in patients with nonalcoholic steatohepatitis (NASH), with reports of increasing risks of endotoxemia and primary bacteremia of gastrointestinal origin [39–41]. In our study, the incidence and risk for overall infection-related hospitalization was not higher among NBNC patients with moderate to markedly elevated liver enzyme levels (presumed NASH) compared with those with normal liver enzymes. However, a higher crude incidence for intra-abdominal infection was noted, but the increased risk was no longer statistically significant after adjustment for important risk factors such as diabetes. Because the study participants were volunteers from a health screening service and were relatively healthy and more health-conscious (only 20% of participants had obesity and had diabetes among NBNC patients with moderate to markedly elevated liver enzyme levels), further studies focusing on high-risk patients are needed to examine the risk of infection among patients with NASH.

In our analyses examining the impact of antiviral therapy on infection risks, patients with HCV who received ribavirin plus peg-interferon therapy, compared to untreated patients with HCV, had reduced risks for hospitalization for all infections, many site-specific infections, and infection-related deaths. Although the apparent negative association among patients treated for HCV can be interpreted as the effect of antiviral therapy on infection risks, other possible explanations may include a better care quality for these patients, because a similar risk reduction also emerged in a comparison of patients with HBV who were treated compared with those who were untreated. Furthermore, although we applied a hd-PS matching technique to control baseline differences between comparison groups, we cannot exclude the possibility that residual confounding factors—such as fewer comorbid diseases or higher education level or socioeconomic status—may also have played a role in the observed association between patients receiving treatment for HCV and having reduced risks for hospitalization for infection and infection-related mortality. Further investigation regarding the effect of antiviral therapy on infection risks for patients with HCV, including the effect of novel DAAs, are needed.

The strength of this study included the use of community-based health screening data from a large number of participants, with long follow-up. Thus, with sufficient information on patient lifestyle and laboratory data, we could extensively evaluate the risk for infection among patients in the different liver disease categories and further explore possible mechanisms of action. With the linkage to the administrative database, prolonged longitudinal follow-up for any clinically significant infection event, with very low missing rate, would be possible.

However, the study had several limitations. First, the definition of HCV infection was based on anti-HCV seropositivity in our study, and it is likely that most patients were chronically infected after their initial HCV exposure. The risk of infection associated with active HCV [42], different HCV subtypes, and viral load needs further investigation [43–45]. Second, although 75.5% and 70.1% of NC-HBV and NC-HCV patients were newly diagnosed with viral hepatitis at this screening service, we did not have information about the number of years that patients had been living with HBV or HCV because the clinical symptoms after infection are usually vague and nonspecific. Third, participants were grouped and analyzed according to baseline information and laboratory data. Because no subsequent data were available during follow-up, we could not exclude the possibility that patients with HCV infection progressed more rapidly to liver cirrhosis than those in other liver disease categories; such a change could have contributed to the increased infection risks. However, in our analysis excluding high-risk patients for liver cirrhosis (APRI ≥ 1.5 at baseline) or focusing on patients with low baseline APRI, we found consistently elevated risks for hospitalization because of infections and a notably increased risk for infection-related death in patients with low APRI among HCV-infected patients, suggesting a mechanism independent of the extent of liver fibrosis. Otherwise, as the Kaplan–Meier curves for overall infection indicate, the separation of curves between NC-HCV and the reference category appeared early after study initiation and persisted until the end. Visualizing a stable risk difference over time implies a minor role of interval fibrosis progression in the increased infection risk among HCV-infected patients, by which a back-loaded curve would be observed. Fourth, the occurrence of any hospitalization for infection, as well as existing comorbidities, was based on ICD-9-CM codes from inpatient and outpatient records; thus, there might be inaccuracies from the diagnosis, coding, or reporting. Nevertheless, the misclassification should be nondifferential and would bias the results toward the null. Fifth, we could not identify the pathogen or the source (e.g., originally from the community or as an opportunistic infection during hospitalization) associated with each infection episode, and the cases of certain site-specific infections were too few to make a precise estimate. Sixth, although we adjusted for crucial variables associated with susceptibility to infection in our analysis, there may be residual confounding from old age, frailty, vaccination status, or poor general health status. Also, we could not exclude the possibility that the higher risk for lower respiratory tract infection may result from a higher prevalence of patients with HCV receiving gastric acid–suppressive agents or who were hospitalized for gastrointestinal bleeding [46]. Seventh, we used self-reported BMI to control for the potential influence of adiposity on infection risk in the present analysis. Studies have suggested that men tend to overreport their heights and women tend to underreport their weights. Meanwhile, BMI cannot distinguish between weight from fat and weight from muscle and bone [47]. Eighth, our results were based on data from a health-screening program in New Taipei City, from which—according to the protocol for enrollment—patients with liver cirrhosis and missing data on baseline or follow-up information were excluded. Because the missing rate was low (6% of missing on baseline and <0.2% on follow-up information) and reasons underlying the missing data were likely to be independent of both exposure categories and the outcome, the bias from missing data should be negligible. However, the volunteers were thought to be relatively healthier and have a higher health literacy. The results might not be generalizable to the general population. Lastly, we could not evaluate the effect of DAA treatment on infection risk based on current data because the study period (2005–2014) pre-dated the NHI reimbursement of DAAs since 2017.

In addition to patients with liver cirrhosis, patients infected with HCV and without cirrhosis had a higher risk for generalized and some site-specific infections. These results suggest that special precautions should be taken when an HCV-infected patient presents with initial symptoms of clinical infection. Furthermore, large-scale population-based studies are needed to evaluate the effectiveness of certain infection-prevention strategies (e.g., preventive antibiotic use, vaccination) or DAA therapy in reducing the risk of infection in this patient population.

## Supporting information

S1 Table ICD-9-CM codes, health insurance reimbursement codes, and ATC codes used in the study. ATC, Anatomical Therapeutic Chemical; ICD-9-CM, International Classification of Diseases, ninth revision, Clinical Modification.
(DOCX)

S2 Table Characteristics of study participants who were excluded, those who were finally included, and the 1 million sample of NHI beneficiaries. (DOCX)

S3 Table Characteristics of the study participants with NC-HBV and NC-HCV infection who received and those who did not receive antiviral therapy. (DOCX)

S4 Table Sensitivity analysis: The association between different liver disease categories and risk of hospitalization for infection syndrome and infection-related mortality compared with NBNC patients with normal to mildly elevated liver enzyme levels adjusted for continuous and categorical BMI, continuous FPG, and continuous eGFR (*N* = 115,336). (DOCX)

S5 Table Sensitivity analysis: The association between different liver disease categories and risk of hospitalization for infection syndrome and infection-related mortality compared with NBNC patients with normal to mildly elevated liver enzyme levels after excluding participants who had the diagnoses of human immunodeficiency virus infection and opioid dependence or abuse, received dialysis, and those with APRI ≥ 1.5 (*N* = 114,307). (DOCX)

S6 Table Sensitivity analysis: The association between different liver disease categories and risk of hospitalization for infection syndrome and infection-related mortality compared with NBNC patients with normal to mildly elevated liver enzyme levels after excluding participants who had the diagnoses of human immunodeficiency virus infection and opioid dependence or abuse, NC-HBV and NC-HCV patients who received antiviral therapy during the study period, and additionally controlled for Charlson comorbidity score (*N* = 114,653). (DOCX)

S7 Table Stratified analysis: The association between NC-HCV stratified on ALT level, APRI, alcohol use, and risk of hospitalization for infection syndrome and infection-related mortality compared with NBNC patients with normal to mildly elevated liver enzyme levels (*N* = 103,630). (DOCX)

S8 Table The association between different liver disease categories and risk of hospitalization for infection syndrome and infection-related mortality compared with NBNC patients with normal to mildly elevated liver enzyme levels in participants aged <50 years (*N* = 50,922). (DOCX)

S9 Table The association between different liver disease categories and risk of hospitalization for infection syndrome and infection-related mortality compared with NBNC patients with normal to mildly elevated liver enzyme levels in participants aged ≥ 50 years (*N* = 64,414). (DOCX)

S10 Table The association between different liver disease categories and risk of hospitalization for infection syndrome and infection-related mortality compared with NBNC patients with normal to mildly elevated liver enzyme levels in men (*N* = 41,005). (DOCX)

S11 Table The association between different liver disease categories and risk of hospitalization for infection syndrome and infection-related mortality compared with NBNC patients with normal to mildly elevated liver enzyme levels in women (*N* = 74,331). (DOCX)

S12 Table Baseline demographics, comorbidities, medication use, and resource utilization, measured within 1 year before the index date among HCV patients who received and those who did not receive antiviral therapy before and after PS matching. (DOCX)

S13 Table Follow-up duration, number of incident cases, and crude incidence of hospitalization for infection syndrome and infection-related mortality among HCV patients who received and those who did not receive antiviral therapy before and after PS and hd-PS matching. (DOCX)

S14 Table Baseline demographics, comorbidities, medication use, and resource utilization, measured within 1 year before the index date among HBV patients who received and those who did not receive antiviral therapy before and after PS and hd-PS matching. (DOCX)

S15 Table Follow-up duration, number of incident cases, and crude incidence of hospitalization for infection syndrome and infection-related mortality among HBV patients who received and those who did not receive antiviral therapy before and after PS and hd-PS matching. (DOCX)

S16 Table Risk of hospitalization for infection syndrome and infection-related mortality comparing HBV patients who received antiviral therapy to those who did not receive antiviral therapy. (DOCX)

S1 Text Study protocol and statistical analysis plan. (DOCX)

S2 Text STROBE statement. Checklist of items that should be included in reports of cohort studies.
(DOC)

## Abbreviations

ALT: alanine aminotransferase
APRI: AST to platelet ratio index
AST: aspartate transaminase
BMI: body mass index
CI: confidence interval
CKD-EPI: Chronic Kidney Disease Epidemiology Collaboration
DAA: direct-acting antiviral agent
eGFR: estimated glomerular filtration rate
FPG: fasting plasma glucose
HBV: hepatitis B virus
HCV: hepatitis C virus
hd-PS: high-dimensional propensity score
HR: hazard ratio
ICD-9-CM: International Classification of Diseases, ninth Revision, Clinical Modification
NAFLD: nonalcoholic fatty liver disease
NASH: nonalcoholic steatohepatitis
NBNC: no HBV or HCV infection
NC-HBV: noncirrhotic with HBV infection
NC-HBVHCV: noncirrhotic with HBV/HCV coinfection
NC-HCV: noncirrhotic with HCV infection
NHIRD: National Health Insurance Research Database
STROBE: Strengthening the Reporting of Observational Studies in Epidemiology
TLR: Toll-like receptor
UNL: upper normal limit

## References

[1] Lee M-H, Yang H-I, Yuan Y, L’Italien G, Chen C-J. Epidemiology and natural history of hepatitis C virus infection. World J Gastroenterol. 2014;20(28):9270–80. 25071320 10.3748/wjg.v20.i28.9270 PMC4110557

[2] Mohamed AA, Elbedewy TA, El-Serafy M, El-Toukhy N, Ahmed W, Ali El Din Z. Hepatitis C virus: A global view. World J Hepatol. 2015;7(26):2676–80. 10.4254/wjh.v7.i26.2676 26609344 PMC4651911

[3] Sievert W, Altraif I, Razavi Homie A, Abdo A, Ahmed Ezzat A, AlOmair A, et al A systematic review of hepatitis C virus epidemiology in Asia, Australia and Egypt. Liver. 2011;31(s2):61–80. 10.1111/j.1478-3231.2011.02540.x 21651703

[4] Sun CA, Chen HC, Lu CF, You SL, Mau YC, Ho MS, et al Transmission of hepatitis C virus in Taiwan: prevalence and risk factors based on a nationwide survey. J Med Virol. 1999;59(3):290–6. 10502258

[5] Bonnel AR, Bunchorntavakul C, Reddy KR. Immune dysfunction and infections in patients with cirrhosis. Clin Gastroenterol Hepatol. 2011;9(9):727–38. 10.1016/j.cgh.2011.02.031 21397731

[6] Bunchorntavakul C, Chamroonkul N, Chavalitdhamrong D. Bacterial infections in cirrhosis: A critical review and practical guidance. World J Hepatol. 2016;8(6):307–21. 10.4254/wjh.v8.i6.307 26962397 PMC4766259

[7] El-Serag HB, Anand B, Richardson P, Rabeneck L. Association between hepatitis C infection and other infectious diseases: a case for targeted screening? Am J Gastroenterol. 2003;98(1):167–74. 12526953 10.1111/j.1572-0241.2003.07176.x

[8] Jirillo E, Greco B, Caradonna L, Satalino R, Pugliese V, Cozzolongo R, et al Evaluation of cellular immune responses and soluble mediators in patients with chronic hepatitis C virus (cHCV) infection. Immunopharmacol Immunotoxicol. 1995;17(2):347–64. 10.3109/08923979509019756 7650295

[9] King E, Trabue C, Yin D, Yao ZQ, Moorman JP. Hepatitis C: the complications of immune dysfunction. Expert Rev Clin Immunol. 2007;3(2):145–57. 10.1586/1744666X.3.2.145 20477104

[10] Della Bella S, Crosignani A, Riva A, Presicce P, Benetti A, Longhi R, et al Decrease and dysfunction of dendritic cells correlate with impaired hepatitis C virus-specific CD4(+) T-cell proliferation in patients with hepatitis C virus infection. Immunology. 2007;121(2):283–92. 10.1111/j.1365-2567.2007.02577.x 17462079 PMC2265942

[11] Dolganiuc A, Garcia C, Kodys K, Szabo G. Distinct Toll-like receptor expression in monocytes and T cells in chronic HCV infection. World J Gastroenterol. 2006;12(8):1198–204. 16534871 10.3748/wjg.v12.i8.1198 PMC4124429

[12] Kim H, Meyer K, Di Bisceglie AM, Ray R. Hepatitis C virus suppresses C9 complement synthesis and impairs membrane attack complex function. J Virol. 2013;87(10):5858–67. 10.1128/JVI.00174-13 23487461 PMC3648158

[13] Marrie TJ, Tyrrell GJ, Majumdar SR, Eurich DT. Concurrent Infection with Hepatitis C Virus and Streptococcus pneumoniae. Emerg Infect Dis. 2017;23(7):1118–23. 10.3201/eid2307.161858 28628455 PMC5512482

[14] Wu PH, Lin YT, Hsieh KP, Chuang HY, Sheu CC. Hepatitis C Virus Infection Is Associated With an Increased Risk of Active Tuberculosis Disease: A Nationwide Population-Based Study. Medicine. 2015;94(33):e1328 10.1097/MD.0000000000001328 26287416 PMC4616441

[15] Laupland KB, Ross T, Gregson DB. Staphylococcus aureus Bloodstream Infections: Risk Factors, Outcomes, and the Influence of Methicillin Resistance in Calgary, Canada, 2000–2006. J Infect Dis. 2008;198(3):336–43. 10.1086/589717 18522502

[16] Awad SS, Elhabash SI, Lee L, Farrow B, Berger DH. Increasing incidence of methicillin-resistant Staphylococcus aureus skin and soft-tissue infections: reconsideration of empiric antimicrobial therapy. Am J Surg. 2007;194(5):606–10. 10.1016/j.amjsurg.2007.07.016 17936421

[17] Miller LG, Perdreau-Remington F, Rieg G, Mehdi S, Perlroth J, Bayer AS, et al Necrotizing fasciitis caused by community-associated methicillin-resistant Staphylococcus aureus in Los Angeles. N Engl J Med. 2005;352(14):1445–53. 10.1056/NEJMoa042683 15814880

[18] Reddy S, Sullivan R, Zaiden R, De Mendoza VL, Naik N, Vega KJ, et al Hepatitis C infection and the risk of bacteremia in hemodialysis patients with tunneled vascular access catheters. South Med J. 2009;102(4):374–7. 10.1097/SMJ.0b013e31819bc34c 19279528

[19] Nakasone H, Kurosawa S, Yakushijin K, Taniguchi S, Murata M, Ikegame K, et al Impact of hepatitis C virus infection on clinical outcome in recipients after allogeneic hematopoietic cell transplantation. Am J Hematol. 2013;88(6):477–84. 10.1002/ajh.23436 23483626

[20] Mizrahi M, Roemi L, Shouval D, Adar T, Korem M, Moses A, et al Bacteremia and "Endotipsitis" following transjugular intrahepatic portosystemic shunting. World J Hepatol. 2011;3(5):130–6. 10.4254/wjh.v3.i5.130 21731907 PMC3124881

[21] Lopez-Medrano F, Fernandez-Ruiz M, Morales JM, San-Juan R, Cervera C, Carratala J, et al Impact of hepatitis C virus infection on the risk of infectious complications after kidney transplantation: data from the RESITRA/REIPI cohort. Transplantation. 2011;92(5):543–9. 10.1097/TP.0b013e318225dbae 21869745

[22] Singh N, Gayowski T, Wagener MM, Marino IR. Increased infections in liver transplant recipients with recurrent hepatitis C virus hepatitis. Transplantation. 1996;61(3):402–6. 10.1097/00007890-199602150-00014 8610350

[23] Scher D, Kanlic E, Bader J, Ortiz M, Abdelgawad A. Hepatitis C viral infection as an associated risk factor for necrotizing fasciitis. Orthopedics. 2012;35(4):e510–3. 10.3928/01477447-20120327-43 22495851

[24] Lee PH, Fu H, Lai TC, Chiang CY, Chan CC, Lin HH. Glycemic Control and the Risk of Tuberculosis: A Cohort Study. PLoS Med. 2016;13(8):e1002072 10.1371/journal.pmed.1002072 27505150 PMC4978445

[25] National Health Insurance Administration Universal Health Coverage in Taiwan. Updated 2018 June 08. https://www.nhi.gov.tw/English/Content_List.aspx?n=8FC0974BBFEFA56D&topn=ED4A30E51A609E49. [cited 2018 Nov 20].

[26] Kramer JR, Davila JA, Miller ED, Richardson P, Giordano TP, El-Serag HB. The validity of viral hepatitis and chronic liver disease diagnoses in Veterans Affairs administrative databases. Aliment Pharmacol Ther. 2007;27(3):274–82. 10.1111/j.1365-2036.2007.03572.x 17996017

[27] Schisterman EF, Cole SR, Platt RW. Overadjustment bias and unnecessary adjustment in epidemiologic studies. Epidemiology. 2009;20(4):488–95. 10.1097/EDE.0b013e3181a819a1 19525685 PMC2744485

[28] Bunchorntavakul C, Chavalitdhamrong D. Bacterial infections other than spontaneous bacterial peritonitis in cirrhosis. World J Hepatol. 2012;4(5):158–68. 10.4254/wjh.v4.i5.158 22662285 PMC3365435

[29] Schneeweiss S, Rassen JA, Glynn RJ, Avorn J, Mogun H, Brookhart MA. High-dimensional propensity score adjustment in studies of treatment effects using health care claims data. Epidemiology. 2009;20(4):512–22. 10.1097/EDE.0b013e3181a663cc 19487948 PMC3077219

[30] Melia MT, Bräu N, Poordad F, Lawitz EJ, Shiffman ML, McHutchison JG, et al Infections During Peginterferon/Ribavirin Therapy Are Associated With the Magnitude of Decline in Absolute Lymphocyte Count: Results of the IDEAL Study. Clin Infect Dis. 2014;58(7):960–9. 10.1093/cid/ciu009 24399086 PMC3968310

[31] Cooper CL, Al-Bedwawi S, Lee C, Garber G. Rate of Infectious Complications during Interferon-Based Therapy for Hepatitis C Is Not Related to Neutropenia. Clin Infect Dis. 2006;42(12):1674–8. 10.1086/504386 16705570

[32] Fried MW. Side effects of therapy of hepatitis C and their management. Hepatology. 2002;36(5 Suppl 1):S237–44. 10.1053/jhep.2002.36810 12407599

[33] Inoue T, Nakayama J, Moriya K, Kawaratani H, Momoda R, Ito K, et al Gut Dysbiosis Associated With Hepatitis C Virus Infection. Clin Infect Dis. 2018;67(6):869–77. 10.1093/cid/ciy205 29718124

[34] Jalan R, Fernandez J, Wiest R, Schnabl B, Moreau R, Angeli P, et al Bacterial infections in cirrhosis: a position statement based on the EASL Special Conference 2013. J Hepatol. 2014;60(6):1310–24. 10.1016/j.jhep.2014.01.024 24530646

[35] Preveden T, Scarpellini E, Milic N, Luzza F, Abenavoli L. Gut microbiota changes and chronic hepatitis C virus infection. Expert Rev Gastroenterol Hepatol. 2017;11(9):813–9. 10.1080/17474124.2017.1343663 28621554

[36] Dominitz JA, Boyko EJ, Koepsell TD, Heagerty PJ, Maynard C, Sporleder JL, et al Elevated prevalence of hepatitis C infection in users of United States veterans medical centers. Hepatology. 2005;41(1):88–96. 10.1002/hep.20502 15619249

[37] Nseir W, Taha H, Khateeb J, Grosovski M, Assy N. Fatty Liver Is Associated with Recurrent Bacterial Infections Independent of Metabolic Syndrome. Dig Dis Sci. 2011;56(11):3328–34. 10.1007/s10620-011-1736-5 21562784

[38] Miura K, Seki E, Ohnishi H, Brenner DA. Role of toll-like receptors and their downstream molecules in the development of nonalcoholic Fatty liver disease. Gastroenterol Res Pract. 2010;2010:362847 10.1155/2010/362847 21274430 PMC3026974

[39] Ghetti FDF, Oliveira DG, Oliveira JMD, De Castro Ferreira Lincoln Eduardo Villela Vieira, Cesar DE, Moreira APB. Influence of gut microbiota on the development and progression of nonalcoholic steatohepatitis. Eur J Nutr. 2017;57(3):861–76. 10.1007/s00394-017-1524-x 28875318

[40] Pang J, Xu W, Zhang X, Wong GL-H, Chan AW-H, Chan H-Y, et al Significant positive association of endotoxemia with histological severity in 237 patients with non-alcoholic fatty liver disease. Aliment Pharmacol Ther. 2017;46(2):175–82. 10.1111/apt.14119 28464257

[41] Nseir W, Artul S, Nasrallah N, Mahamid M. The association between primary bacteremia of presumed gastrointestinal origin and nonalcoholic fatty liver disease. Ital J Gastroenterol Hepatol. 2016;48(3):343–4. 10.1016/j.dld.2015.10.004 26534773

[42] Kaka AS, Filice GA, Kuskowski M, Musher DM. Does active hepatitis C virus infection increase the risk for infection due to Staphylococcus aureus? Eur J Clin Microbiol. 2017;36(7):1217–23. 10.1007/s10096-017-2912-0 28160146

[43] Li W, Jin R, Chen P, Zhao G, Li N, Wu H. Clinical correlation between HBV infection and concomitant bacterial infections. Sci Rep. 2015;5:15413 10.1038/srep15413 26634436 PMC4669448

[44] Nahon P, Bourcier V, Layese R, Audureau E, Cagnot C, Marcellin P, et al Eradication of Hepatitis C Virus Infection in Patients With Cirrhosis Reduces Risk of Liver and Non-Liver Complications. Gastroenterology. 2017;152(1):142–56.e2. 10.1053/j.gastro.2016.09.009 27641509

[45] Nahon P, Lescat M, Layese R, Bourcier V, Talmat N, Allam S, et al Bacterial infection in compensated viral cirrhosis impairs 5-year survival (ANRS CO12 CirVir prospective cohort). Gut. 2017;66(2):330–41. 10.1136/gutjnl-2015-310275 26511797

[46] Eom C-S, Jeon CY, Lim J-W, Cho E-G, Park SM, Lee K-S. Use of acid-suppressive drugs and risk of pneumonia: a systematic review and meta-analysis. CMAJ. 2011;183(3):310–9. 10.1503/cmaj.092129 21173070 PMC3042441

[47] Romero-Corral A, Somers VK, Sierra-Johnson J, Thomas RJ, Collazo-Clavell ML, Korinek J, et al Accuracy of body mass index in diagnosing obesity in the adult general population. Int J Obes. 2008;32(6):959–66. 10.1038/ijo.2008.11 PMC2877506 18283284

