# Supplementary material for: Incidence of hospitalization for infection among patients with hepatitis B or C virus infection without cirrhosis in Taiwan: A cohort study
Source: PLoS Med. 2019 Sep 13;16(9):e1002894. doi: 10.1371/journal.pmed.1002894 (PMC6743759; doi:10.1371/journal.pmed.1002894)
Supplement: S1 Table — ATC, Anatomical Therapeutic Chemical; ICD-9-CM, International Classification of Diseases, ninth revision, Clinical Modification. (DOCX) [file pmed.1002894.s001.docx]

**S1 Table. ICD-9-CM codes, health insurance reimbursement codes, and ATC codes used in the study. ATC, Anatomical Therapeutic Chemical; ICD-9-CM, International Classification of Diseases, ninth revision, Clinical Modification.**

| **Infection syndrome** |  |
| --- | --- |
| Septicemia | 038, 041.9, 790.7, 785.52 |
| Lower respiratory tract infection | 480, 487, 481, 482, 483, 485, 486, 510, 513 |
| Intra-abdominal infection | 540, 541, 542, 562.01, 562.03, 562.11, 562.13, 566, 567, 569.5, 572.0, 572.1, 575.0 |
| Reproductive and urinary tract infection | 590, 599.0, 601, 604, 614, 615, 616 |
| Skin and soft tissue infection | 680, 681, 682, 683, 684, 685, 686 |
| Osteomyelitis | 711.0, 730 |
| Necrotizing fasciitis | 728.86 |
| Infectious intestinal diseases | 001-009 |
| **Comorbidities** |  |
| Liver cirrhosis | 571.2, 571.5, 571.6 |
| Dialysis | ICD-9-CM codes: V451, V560, V568  Health insurance reimbursement codes: 58001C, 58001CA, 58002C, 58002CB, 58009B, 58010A, 58010B, 58011A, 58011AB, 58011B, 58011C, 58012A, 58012B, 58013C, 58014C, 58017B, 58017C, 58018C, 58019C, 58020C, 58021C, 58022C, 58023C, 58024C, 58025C, 58026C, 58027C, 58028C, 58029C, 58030B, 69006C, 69006C, CGDW1960012C, CGS01440042C, CGS01440132C, CKD006752L2X, CKD006753L2X, CKDD1135132C, CKDD1170052C, CKDD117005KD, CKDD1170092C, CKDD117009KD, CKDD122334BQ, CKDD124334BQ, CKDD1390032C, CKDD139003KD, CKDD154334BQ, CKDD1881CK2C, CKDD1881SK2C, CKDD1883CK2C, CKDD1883SK2C, CKDD210598SB, CKDD2140012C, CKDD214001KD, CKDD2140022C, CKDD214002KD, CKDD214554NL, CKDD2330742C, CKDD2420052C, CKDD242005KD, CKDD2450072C, CKDD245007KD, CKDD245540SB, CKDD245542SB, CKDD2480012C, CKDD257331BA, CKDD2690012C, CKDD269001KD, CKDD269033BA, CKDD2881452C, CKDD288155KD, CKDD2SL12PM0, CKDD2SL18PM0, CKDD323334BQ, CKDD3456032C, CKDD345603KD, CKP010T2022C, CKP018812N2C, CKP018812NKD, CKP018814N2C, CKP018814NKD, CKP018817N2C, CKP018817NKD, CKP018880N2C, CKP018880NKD, CKP018884N2C, CKP018888N2C, CKP018890N2C, CKP018890NKD, CKP022C410BT, CKP0413836FA, CKP04C4407BT, CKP04C4479BT, CKP04C8303BT, CPC0260048DV, FUK0500M60GA, FUK050M100GA, FUK05ST100GA, FUK05ST60PGA, FUK05TS404FN, FUK05TS413FN, HEF03PRFPEGA, HEF03PRLPEGA, HEF03PRTPEGA |
| Hypertension | 401-404 |
| Ischemic heart disease | 411, 413, 414 |
| Myocardial infarction | 410, 412 |
| Cardiac dysrhythmia/atrial fibrillation | 427 |
| Congestive heart failure | 428, 398.91, 402.01, 402.11, 402.91, 404.01, 404.11, 404.91, 404.03, 404.13, 404.93 |
| Stroke | 430, 431, 432, 433, 434, 436 |
| Peripheral vascular disease | 440.2, 440.4, 443.81, 443.9 |
| Disorders of lipid metabolism | 272 |
| Chronic lung disease | 490-496, 500-508 |
| Autoimmune disease | 710.2, 696.0, 696.1, 714.0, 710.0, 710.1, 710.4, 283.0, 245.2, 340, 358.0, 364.0, 364.3, 555, 556 |
| Dementia | 290.0-290.4, 291.2, 294.1, 331.0-331.2, 290.10-290.13, 290.20, 290.21, 290.40-290.43, 294.10, 294.11, 331.11, 331.19, 331.82 |
| Cancer | 140-208 |
| Human immunodeficiency virus infection | 042 |
| Opioid dependence or abuse | 304.0, 304.7, 305.5 |
| Peptic ulcer disease | 531-535, 578.0, 578.1, 578.9 |
| Codes that used in the analyses that examined the impact of antiviral treatment on the risks for infectious diseases morbidity |  |
| Hepatitis C virus infection | 07041, 07044, 07051, 07054, 07070, 07071, V0262 |
| Hepatitis B virus infection | 0702, 07020, 07021, 07022, 07023, 0703, 07030, 07031, 07032, 07033, 07042, 07052, V0261 |
| **Medications (ATC codes)** |  |
| Anti-diabetes medications | A10 |
| Systemic steroids | H02AA02, H02AB02, H02AB04, H02AB06, H02AB10, H02BX, H02BX91 |
| Proton pump inhibitor or H2-receptor blocker | A02BA, A02BC |
| Anti-viral medications for viral hepatitis C and B | L03AB04, L03AB05, L03AB11, L03AB10, J05AF05, J05AF08, J05AF10, J05AF11, J05AF07, J05AB04 |
