# Supplementary material for: Incidence of hospitalization for infection among patients with hepatitis B or C virus infection without cirrhosis in Taiwan: A cohort study
Source: PLoS Med. 2019 Sep 13;16(9):e1002894. doi: 10.1371/journal.pmed.1002894 (PMC6743759; doi:10.1371/journal.pmed.1002894)
Supplement: S2 Table — (DOCX) [file pmed.1002894.s002.docx]

**S2 Table. Characteristics of study participants who were excluded, those who were finally included, and the 1 million sample of NHI beneficiaries.**

|  | Participants of New Taipei City Health Screening in 2005-2008 finally included | Participants of New Taipei City Health Screening in 2005-2008 excluded | One million sample of NHI beneficiaries |
| --- | --- | --- | --- |
| Number | 115,336 | 7,552 | 756,830 |
| Male (%) | 35.55 | 35.86 | 47.90 |
| Age, mean (SD) | 52.22 (11.74) | 45.19 (13.17) | 45.48 (16.55) |
| 20-40 | 15.78 | 52.08 | 43.41 |
| 41-50 | 31.85 | 16.75 | 20.57 |
| 51-60 | 29.20 | 16.39 | 17.59 |
| 61-70 | 15.34 | 9.14 | 9.20 |
| 71-100 | 7.82 | 5.64 | 9.23 |
| **Comorbidities, %** |  |  |  |
| Diabetes mellitus | 7.59 | 5.08 | 7.66 |
| Hypertension | 18.74 | 12.35 | 16.43 |
| Ischemic heart disease | 6.00 | 4.25 | 4.64 |
| Myocardial infarction | 0.22 | 0.17 | 0.28 |
| Cardiac dysrhythmia/atrial fibrillation | 2.73 | 2.34 | 2.38 |
| Congestive heart failure | 1.33 | 1.10 | 1.45 |
| Stroke | 1.38 | 1.24 | 1.73 |
| Peripheral vascular disease | 0.44 | 0.25 | 0.53 |
| Disorders of lipid metabolism | 11.88 | 8.61 | 9.77 |
| Chronic lung disease | 7.68 | 6.48 | 6.55 |
| Chronic kidney disease | 0.34 | 0.24 | 0.91 |
| Autoimmune disease | 2.70 | 2.37 | 2.37 |
| Dementia | 0.22 | 0.24 | 0.73 |
| Cancer | 1.87 | 1.52 | 2.29 |
| Charlson comorbidity score | 0.42 (0.87) | 0.34 (0.79) | 0.43 (1.00) |
| Human immunodeficiency virus infection | <0.01 | 0.00 | 0.05 |
| Opioid dependence or abuse | <0.01* | 0.00 | 0.02 |

*The exact case numbers in either category were too small to be retrieved because of the authority’s policy regulation.

Abbreviations:ALT, alanine aminotransferase; APRI, AST to Platelet Ratio Index; AST, aspartate transaminase; SD, standard deviation; NHI, National Health Insurance.
