# Supplementary material for: Incidence of hospitalization for infection among patients with hepatitis B or C virus infection without cirrhosis in Taiwan: A cohort study
Source: PLoS Med. 2019 Sep 13;16(9):e1002894. doi: 10.1371/journal.pmed.1002894 (PMC6743759; doi:10.1371/journal.pmed.1002894)
Supplement: S3 Table — (DOCX) [file pmed.1002894.s003.docx]

**S3 Table. Characteristics of the study participants with NC-HBV and NC-HCV infection who received and those who did not receive antiviral therapy.**

|  | NC-HBV | | NC-HCV | |
| --- | --- | --- | --- | --- |
|  | who received anti-viral therapy | who did not receive anti-viral therapy | who received anti-viral therapy | who did not receive anti-viral therapy |
| Number | 339 | 7977 | 339 | 2500 |
| Male (%) | 50.15 | 39.51 | 41.59 | 33.56 |
| Age, mean (SD) | 49.51 (10.27) | 49.70 (10.93) | 52.74 (8.80) | 57.62 (12.11) |
| 20-40 | 19.47 | 20.66 | 9.44 | 7.28 |
| 41-50 | 37.76 | 36.40 | 28.91 | 22.84 |
| 51-60 | 28.61 | 26.49 | 41.00 | 29.88 |
| 61-70 | 11.21 | 11.80 | 18.88 | 23.76 |
| 71-100 | 2.95 | 4.65 | 1.77 | 16.24 |
| BMI (%) |  |  |  |  |
| Underweight | 2.65 | 3.15 | 1.47 | 3.44 |
| Normal | 59.00 | 58.84 | 56.34 | 56.84 |
| Overweight | 32.74 | 31.55 | 34.22 | 32.72 |
| Obesity | 5.60 | 6.46 | 7.96 | 7.00 |
| Cigarette smoking (%) |  |  |  |  |
| Never | 78.17 | 76.97 | 74.63 | 76.64 |
| Quitted | 7.96 | 6.66 | 9.44 | 5.88 |
| Current | 13.86 | 16.37 | 15.93 | 17.48 |
| Alcohol consumption (%) |  |  |  |  |
| Never | 63.72 | 59.87 | 64.60 | 69.00 |
| Quitted | 1.77 | 1.98 | 6.19 | 3.24 |
| Regular | 5.90 | 7.52 | 7.37 | 6.44 |
| Sometimes | 28.61 | 30.63 | 21.83 | 21.32 |
| Education level (%) |  |  |  |  |
| Illiterate | 4.42 | 6.76 | 9.44 | 18.36 |
| Literate but not attending elementary school | 1.47 | 1.63 | 3.54 | 4.20 |
| Elementary school | 19.76 | 22.55 | 33.04 | 34.08 |
| Junior high school | 15.93 | 17.93 | 20.65 | 16.08 |
| High school | 34.22 | 30.32 | 20.65 | 18.36 |
| College | 20.94 | 18.94 | 11.50 | 8.32 |
| Graduate school | 3.24 | 1.87 | 1.18 | 0.60 |
| **Laboratory data (mean, SD)** |  |  |  |  |
| Albumin, g/dL (mean, SD) | 4.59 (0.30) | 4.62 (0.26) | 4.53 (0.28) | 4.51 (0.28) |
| AST, U/L (mean, SD) | 41.75 (45.16) | 27.29 (17.25) | 59.41 (50.80) | 35.45 (28.08) |
| ALT, U/L (mean, SD) | 57.04 (80.78) | 29.20 (25.06) | 78.94 (78.59) | 37.92 (41.90) |
| APRI score (mean, SD) | 0.47 (0.57) | 0.28 (0.24) | 0.71 (0.73) | 0.40 (0.44) |
| APRI category (%) |  |  |  |  |
| < 0.5 | 76.99 | 93.96 | 54.28 | 81.12 |
| 0.5-<1 | 16.52 | 4.99 | 27.14 | 13.72 |
| 1-<1.5 | 2.36 | 0.44 | 7.37 | 2.68 |
| 1.5-<2 | 1.18 | 0.35 | 5.90 | 1.08 |
| ≥2 | 0.29 | | 5.31 | 1.28 |
| Fasting plasma glucose, mg/dL (%) |  |  |  |  |
| ≤ 90 | 0.00 | 0.15 | 0.00 | 0.40 |
| 91-130 | 1.47 | 1.54 | 3.83 | 2.72 |
| 131-200 | 5.01 | 3.50 | 3.54 | 6.72 |
| > 200 | 2.65 | 1.74 | 2.06 | 2.68 |
| eGFR, ml/min/1.73m^2^ (%) |  |  |  |  |
| ≥ 90 | 57.23 | 59.21 | 50.15 | 44.76 |
| 60-89 | 39.23 | 37.18 | 44.84 | 45.28 |
| <59 or on dialysis therapy | 3.54 | 3.61 | 5.01 | 9.96 |

**Abbreviations: ALT, alanine aminotransferase; APRI, AST to Platelet Ratio Index; AST, aspartate transaminase; BMI, body mass index; eGFR, estimated glomerular filtration rate; NC-HBV, noncirrhotic with HBV infection; NC-HCV, noncirrhotic with HCV infection;** **SD, standard deviation.**
