# Supplementary material for: Incidence of hospitalization for infection among patients with hepatitis B or C virus infection without cirrhosis in Taiwan: A cohort study
Source: PLoS Med. 2019 Sep 13;16(9):e1002894. doi: 10.1371/journal.pmed.1002894 (PMC6743759; doi:10.1371/journal.pmed.1002894)
Supplement: S4 Table — (DOCX) [file pmed.1002894.s004.docx]

**S4 Table. Sensitivity analysis: the association between different liver disease categories and risk of hospitalization for infection syndrome and infection-related mortality compared with NBNC patients with normal to mildly elevated liver enzyme levels adjusted for continuous and categorical BMI, continuous FPG, and continuous eGFR (N = 115,336).**

|  | NBNC  ALT normal to 1.5x UNL | NBNC  ALT ≥ 1.5x UNL | | NC-HBV | | NC-HCV | |
| --- | --- | --- | --- | --- | --- | --- | --- |
|  | HR | Adjusted  HR* | Adjusted  HR^†^ | Adjusted  HR* | Adjusted  HR^†^ | Adjusted  HR* | Adjusted  HR^†^ |
| **Hospitalization for infection** |  |  |  |  |  |  |  |
| All infections | 1.0 (Reference) | 1.05 (0.95-1.16) | 1.04 (0.94-1.14) | 0.93 (0.87-1.00) | 0.94 (0.88-1.01) | 1.21 (1.11-1.32) | 1.22 (1.12-1.33) |
| Septicemia | 1.0 (Reference) | 0.98 (0.76-1.26) | 0.96 (0.75-1.23) | 0.88 (0.74-1.05) | 0.89 (0.75-1.06) | 1.24 (1.03-1.51) | 1.27 (1.05-1.53) |
| Lower respiratory tract | 1.0 (Reference) | 1.07 (0.87-1.32) | 1.02 (0.82-1.25) | 0.89 (0.77-1.03) | 0.90 (0.78-1.04) | 1.25 (1.08-1.46) | 1.26 (1.09-1.46) |
| Intra-abdominal | 1.0 (Reference) | 1.26 (1.00-1.59) | 1.26 (1.00-1.59) | 0.81 (0.66-0.98) | 0.81 (0.67-0.98) | 1.22 (0.95-1.57) | 1.22 (0.95-1.58) |
| Reproductive and urinary tract | 1.0 (Reference) | 0.97 (0.82-1.14) | 0.96 (0.81-1.13) | 0.99 (0.89-1.10) | 1.00 (0.90-1.11) | 1.26 (1.11-1.44) | 1.28 (1.12-1.46) |
| Skin and soft tissue | 1.0 (Reference) | 0.87 (0.67-1.14) | 0.89 (0.68-1.16) | 0.96 (0.80-1.16) | 0.97 (0.80-1.17) | 0.91 (0.70-1.19) | 0.92 (0.71-1.21) |
| Osteomyelitis | 1.0 (Reference) | 1.00 (0.49-2.05) | 1.02 (0.50-2.09) | 0.65 (0.36-1.20) | 0.66 (0.36-1.20) | 0.92 (0.47-1.81) | 0.95 (0.49-1.86) |
| Necrotizing fasciitis | 1.0 (Reference) | 0.46 (0.06-3.38) | 0.42 (0.06-3.11) | 0.84 (0.26-2.71) | 0.88 (0.27-2.86) | 1.22 (0.36-4.06) | 1.58 (0.49-5.13) |
| Infectious intestinal diseases | 1.0 (Reference) | 0.95 (0.50-1.79) | 0.89 (0.47-1.69) | 0.83 (0.54-1.28) | 0.83 (0.54-1.28) | 1.38 (0.83-2.28) | 1.38 (0.83-2.28) |
| **Infection-related deaths** | 1.0 (Reference) | 1.64 (0.90-3.01) | 1.52 (0.83-2.78) | 0.85 (0.52-1.39) | 0.86 (0.53-1.40) | 1.38 (0.92-2.06) | 1.38 (0.92-2.06) |

* Adjusted for age (continuous), sex, BMI (continuous), smoking (current, non-current), alcohol consumption, education level, fasting plasma glucose (continuous), eGFR (continuous), systemic steroids use >30 days before study entry, and history of hospitalization within 6 months before hospitalization for infection syndrome.

† Adjusted for continuous age, sex, BMI category, smoking (current, non-current), alcohol consumption, education level, DM (no, fasting glucose ≤130, 131-200, >200), eGFR category, systemic steroids use >30 days before study entry, and history of hospitalization within 6 months before hospitalization for infection syndrome.

**Abbreviations: ALT, alanine aminotransferase; BMI, body mass index; eGFR, estimated glomerular filtration rate; FPG, fasting plasma glucose; HR, hazard ratio; NBNC, no HBV or HCV infection; NC-HBV, noncirrhotic with HBV infection; NC-HCV, noncirrhotic with HCV infection;** **UNL, upper normal limit**
