# Supplementary material for: Incidence of hospitalization for infection among patients with hepatitis B or C virus infection without cirrhosis in Taiwan: A cohort study
Source: PLoS Med. 2019 Sep 13;16(9):e1002894. doi: 10.1371/journal.pmed.1002894 (PMC6743759; doi:10.1371/journal.pmed.1002894)
Supplement: S5 Table — (DOCX) [file pmed.1002894.s005.docx]

**S5 Table. Sensitivity analysis: the association between different liver disease categories and risk of hospitalization for infection syndrome and infection-related mortality compared with NBNC patients with normal to mildly elevated liver enzyme levels after excluding participants who had the diagnoses of human immunodeficiency virus infection and opioid dependence or abuse, received dialysis, and those with APRI ≥ 1.5 (N = 114,307).**

|  | NBNC  ALT normal to 1.5x UNL | NBNC  ALT ≥ 1.5x UNL | | NC-HBV | | NC-HCV | |
| --- | --- | --- | --- | --- | --- | --- | --- |
|  | HR | Adjusted HR* | Adjusted HR^†^ | Adjusted HR* | Adjusted HR^†^ | Adjusted HR* | Adjusted HR^†^ |
| **Hospitalization for infection** |  |  |  |  |  |  |  |
| All infections | 1.0 (Reference) | 1.03 (0.93-1.14) | 1.02 (0.92-1.13) | 0.95 (0.88-1.02) | 0.95 (0.88-1.02) | 1.21 (1.11-1.32) | 1.16 (1.07-1.27) |
| Septicemia | 1.0 (Reference) | 0.97 (0.75-1.25) | 0.91 (0.69-1.19) | 0.89 (0.74-1.07) | 0.90 (0.74-1.08) | 1.22 (1.00-1.49) | 1.19 (0.97-1.46) |
| Lower respiratory tract | 1.0 (Reference) | 1.00 (0.81-1.24) | 0.97 (0.78-1.21) | 0.90 (0.78-1.04) | 0.90 (0.78-1.04) | 1.27 (1.09-1.48) | 1.21 (1.04-1.42) |
| Intra-abdominal | 1.0 (Reference) | 1.27 (1.01-1.60) | 1.29 (1.02-1.64) | 0.82 (0.68-1.00) | 0.82 (0.68-1.00) | 1.21 (0.93-1.57) | 1.22 (0.93-1.58) |
| Reproductive and urinary tract | 1.0 (Reference) | 0.95 (0.81-1.12) | 0.96 (0.81-1.13) | 1.00 (0.90-1.12) | 1.01 (0.90-1.12) | 1.27 (1.11-1.45) | 1.24 (1.08-1.42) |
| Skin and soft tissue | 1.0 (Reference) | 0.86 (0.65-1.12) | 0.81 (0.61-1.08) | 0.99 (0.82-1.19) | 0.96 (0.79-1.16) | 0.92 (0.70-1.21) | 0.85 (0.64-1.14) |
| Osteomyelitis | 1.0 (Reference) | 1.02 (0.50-2.07) | 0.93 (0.44-1.99) | 0.67 (0.37-1.23) | 0.62 (0.33-1.16) | 0.88 (0.43-1.78) | 0.68 (0.30-1.53) |
| Necrotizing fasciitis | 1.0 (Reference) | 0.45 (0.06-3.29) | 0.47 (0.06-3.50) | 0.93 (0.29-3.02) | 0.94 (0.29-3.05) | 1.09 (0.26-4.54) | 1.12 (0.27-4.64) |
| Infectious intestinal diseases | 1.0 (Reference) | 0.90 (0.48-1.70) | 0.94 (0.50-1.77) | 0.81 (0.52-1.26) | 0.82 (0.52-1.27) | 1.40 (0.84-2.31) | 1.36 (0.81-2.28) |
| **Infection-related deaths** | 1.0 (Reference) | 1.48 (0.78-2.81) | 1.45 (0.74-2.84) | 0.64 (0.35-1.16) | 0.65 (0.36-1.19) | 1.34 (0.88-2.05) | 1.27 (0.82-1.97) |

* Excluding participants who had the diagnoses of human immunodeficiency virus infection and opioid dependence or abuse (N = 5) and received dialysis (N = 635); adjusted for continuous age, sex, BMI category, smoking (current, non-current), alcohol consumption, education level, DM (no, fasting glucose ≤130, 131-200, >200), eGFR category, systemic steroids use >30 days before study entry, and history of hospitalization within 6 months before hospitalization for infection syndrome.

† Excluding participants who had the diagnoses of human immunodeficiency virus infection and opioid dependence or abuse (N = 5), received dialysis (N = 635), and those with no AST to Platelet Ratio Index (APRI) data (N = 92) or APRI ≥ 1.5 (N=297); adjusted for continuous age, sex, BMI category, smoking (current, non-current), alcohol consumption, education level, DM (no, fasting glucose ≤130, 131-200, >200), eGFR category, systemic steroids use >30 days before study entry, and history of hospitalization within 6 months before hospitalization for infection syndrome.

**Abbreviations: ALT, alanine aminotransferase; APRI, AST to platelet ratio index; HR, hazard ratio; NBNC, no HBV or HCV infection; NC-HBV, noncirrhotic with HBV infection; NC-HCV, noncirrhotic with HCV infection; UNL, upper normal limit**
