# Supplementary material for: Incidence of hospitalization for infection among patients with hepatitis B or C virus infection without cirrhosis in Taiwan: A cohort study
Source: PLoS Med. 2019 Sep 13;16(9):e1002894. doi: 10.1371/journal.pmed.1002894 (PMC6743759; doi:10.1371/journal.pmed.1002894)
Supplement: S7 Table — (DOCX) [file pmed.1002894.s007.docx]

**S7 Table. Stratified analysis: the association between NC-HCV stratified on ALT level, APRI, alcohol use, and risk of hospitalization for infection syndrome and infection-related mortality compared with NBNC patients with normal to mildly elevated liver enzyme levels (N = 103,630).**

|  | NBNC  ALT normal to 1.5x UNL | ALT normal - 1.5x UNL  (N= 2,348) | ALT ≥ 1.5x UNL  (N= 491) | Low APRI  (< median§)  (N= 1,420) | High APRI  (≥ median§)  (N= 1,416) | Never alcohol use  (N= 1,944) | Ever alcohol use  (N= 895) |
| --- | --- | --- | --- | --- | --- | --- | --- |
|  | HR | Adjusted  HR* | Adjusted  HR* | Adjusted  HR^†^ | Adjusted  HR^†^ | Adjusted  HR^‡^ | Adjusted  HR^‡^ |
| **Hospitalization for infection** |  |  |  |  |  |  |  |
| All infections | 1.0 (Reference) | 1.18 (1.08-1.29) | 1.48 (1.22-1.81) | 1.18 (1.04-1.34) | 1.26 (1.13-1.41) | 1.26 (1.14-1.39) | 1.14 (0.97-1.35) |
| Septicemia | 1.0 (Reference) | 1.22 (0.99-1.51) | 1.55 (0.98-2.47) | 1.32 (1.00-1.75) | 1.22 (0.95-1.58) | 1.41 (1.14-1.74) | 0.89 (0.57-1.38) |
| Lower respiratory tract | 1.0 (Reference) | 1.17 (0.99-1.39) | 1.81 (1.30-2.53) | 1.18 (0.93-1.50) | 1.32 (1.09-1.59) | 1.32 (1.11-1.56) | 1.13 (0.84-1.52) |
| Intra-abdominal | 1.0 (Reference) | 1.18 (0.89-1.56) | 1.48 (0.86-2.56) | 0.90 (0.59-1.37) | 1.53 (1.12-2.09) | 1.21 (0.89-1.65) | 1.26 (0.83-1.93) |
| Reproductive and urinary tract | 1.0 (Reference) | 1.29 (1.12-1.49) | 1.22 (0.84-1.75) | 1.31 (1.09-1.57) | 1.27 (1.05-1.52) | 1.34 (1.15-1.55) | 1.13 (0.85-1.50) |
| Skin and soft tissue | 1.0 (Reference) | 0.85 (0.63-1.15) | 1.32 (0.75-2.33) | 0.81 (0.53-1.23) | 1.03 (0.73-1.45) | 0.75 (0.53-1.07) | 1.34 (0.89-2.02) |
| Osteomyelitis | 1.0 (Reference) | 0.74 (0.33-1.68) | 2.30 (0.73-7.19) | 0.95 (0.35-2.57) | 0.97 (0.40-2.35) | 1.01 (0.47-2.17) | 0.83 (0.21-3.35) |
| Necrotizing fasciitis | 1.0 (Reference) | 1.89 (0.58-6.16) | NA | 2.35 (0.57-9.76) | 0.97 (0.13-7.09) | 0.78 (0.11-5.69) | 3.44 (0.82-14.46) |
| Infectious intestinal diseases | 1.0 (Reference) | 1.52 (0.90-2.56) | 0.56 (0.08-4.02) | 1.83 (0.97-3.43) | 0.97 (0.43-2.19) | 1.99 (1.20-3.31) | NA |
| **Infection-related deaths** | 1.0 (Reference) | 1.37 (0.90-2.08) | 1.15 (0.29-4.63) | 2.23 (1.35-3.69) | 0.82 (0.44-1.55) | 1.48 (0.96-2.28) | 0.80 (0.26-2.51) |

*Adjusted for continuous age, sex, BMI category, smoking (current, non-current), alcohol consumption, education level, DM (no, fasting glucose ≤130, 131-200, >200), eGFR category, systemic steroids use >30 days before study entry, and history of hospitalization within 6 months before hospitalization for infection syndrome

**†**Adjusted for continuous age, sex, BMI category, smoking (current, non-current), alcohol consumption, education level, DM (no, fasting glucose ≤130, 131-200, >200), eGFR category, systemic steroids use >30 days before study entry, and history of hospitalization within 6 months before hospitalization for infection syndrome

‡ Adjusted for continuous age, sex, BMI category, smoking (current, non-current), education level, DM (no, fasting glucose ≤130, 131-200, >200), eGFR category, systemic steroids use >30 days before study entry, and history of hospitalization within 6 months before hospitalization for infection syndrome.

§ median APRI=0.30

**Abbreviations: ALT, alanine aminotransferase; APRI, AST to platelet ratio index; BMI, body mass index; eGFR, estimated glomerular filtration rate; HR, hazard ratio; NBNC, no HBV or HCV infection; NC-HBV, noncirrhotic with HBV infection; NC-HCV, noncirrhotic with HCV infection; UNL, upper normal limit**
