# Supplementary material for: Incidence of hospitalization for infection among patients with hepatitis B or C virus infection without cirrhosis in Taiwan: A cohort study
Source: PLoS Med. 2019 Sep 13;16(9):e1002894. doi: 10.1371/journal.pmed.1002894 (PMC6743759; doi:10.1371/journal.pmed.1002894)
Supplement: S8 Table — (DOCX) [file pmed.1002894.s008.docx]

**S8 Table. The association between different liver disease categories and risk of hospitalization for infection syndrome and infection-related mortality compared with NBNC patients with normal to mildly elevated liver enzyme levels in participants aged <50 years (N = 50,922).**

|  | NBNC  ALT normal to 1.5x UNL | NBNC  ALT ≥ 1.5x UNL | | NC-HBV | | NC-HCV | |
| --- | --- | --- | --- | --- | --- | --- | --- |
|  | HR | Crude HR | Adjusted HR* | Crude HR | Adjusted HR* | Crude HR | Adjusted HR* |
| **Hospitalization for infection** |  |  |  |  |  |  |  |
| All infections | 1.0 (Reference) | 1.22 (1.03-1.44) | 1.05 (0.88-1.25) | 0.90 (0.80-1.01) | 0.90 (0.80-1.02) | 1.34 (1.06-1.68) | 1.02 (0.81-1.28) |
| Septicemia | 1.0 (Reference) | 1.20 (0.70-2.06) | 0.88 (0.51-1.53) | 0.77 (0.51-1.14) | 0.74 (0.49-1.11) | 2.31 (1.33-4.02) | 1.43 (0.82-2.52) |
| Lower respiratory tract | 1.0 (Reference) | 0.85 (0.50-1.46) | 0.75 (0.44-1.30) | 0.81 (0.59-1.13) | 0.80 (0.57-1.11) | 1.13 (0.58-2.19) | 0.71 (0.37-1.39) |
| Intra-abdominal | 1.0 (Reference) | 1.82 (1.32-2.51) | 1.27 (0.91-1.77) | 0.85 (0.64-1.12) | 0.80 (0.60-1.06) | 1.31 (0.77-2.23) | 1.07 (0.63-1.82) |
| Reproductive and urinary tract | 1.0 (Reference) | 0.96 (0.73-1.26) | 1.15 (0.87-1.52) | 0.93 (0.79-1.10) | 0.99 (0.84-1.18) | 1.34 (0.97-1.87) | 1.12 (0.80-1.56) |
| Skin and soft tissue | 1.0 (Reference) | 1.42 (0.89-2.25) | 0.76 (0.47-1.22) | 1.13 (0.82-1.55) | 1.05 (0.76-1.44) | 1.38 (0.71-2.68) | 0.94 (0.48-1.84) |
| Osteomyelitis | 1.0 (Reference) | 1.36 (0.33-5.65) | 0.61 (0.14-2.67) | 0.67 (0.21-2.17) | 0.58 (0.18-1.90) | 1.39 (0.19-10.1) | 0.60 (0.08-4.65) |
| Necrotizing fasciitis | 1.0 (Reference) | NA | NA | 1.98 (0.23-17.0) | 3.15 (0.33-30.2) | NA | NA |
| Infectious intestinal diseases | 1.0 (Reference) | 1.95 (0.91-4.20) | 1.42 (0.63-3.17) | 0.75 (0.36-1.54) | 0.72 (0.35-1.49) | 0.57 (0.08-4.09) | 0.42 (0.06-3.04) |
| **Infection-related deaths** | 1.0 (Reference) | NA | NA | 1.03 (0.13-8.17) | 1.20 (0.15-9.91) | NA | NA |

*Adjusted for continuous age, sex, BMI category, smoking (current, non-current), alcohol consumption, education level, diabetes (no, fasting glucose ≤130, 131-200, >200), eGFR category, systemic steroids use >30 days before study entry, and history of hospitalization within 6 months before hospitalization for infection syndrome.

**Abbreviations: ALT, alanine aminotransferase; BMI, body mass index; eGFR, estimated glomerular filtration rate; HR, hazard ratio; NA, not applicable; NBNC, no HBV or HCV infection; NC-HBV, noncirrhotic with HBV infection; NC-HCV, noncirrhotic with HCV infection;** **UNL, upper normal limit**
