# Supplementary material for: Incidence of hospitalization for infection among patients with hepatitis B or C virus infection without cirrhosis in Taiwan: A cohort study
Source: PLoS Med. 2019 Sep 13;16(9):e1002894. doi: 10.1371/journal.pmed.1002894 (PMC6743759; doi:10.1371/journal.pmed.1002894)
Supplement: S9 Table — (DOCX) [file pmed.1002894.s009.docx]

**S9 Table. The association between different liver disease categories and risk of hospitalization for infection syndrome and infection-related mortality compared with NBNC patients with normal to mildly elevated liver enzyme levels in participants aged ≥ 50 years (N = 64,414).**

|  | NBNC  ALT normal to 1.5x UNL | NBNC  ALT ≥ 1.5x UNL | | NC-HBV | | NC-HCV | |
| --- | --- | --- | --- | --- | --- | --- | --- |
|  | HR | Crude HR | Adjusted HR* | Crude HR | Adjusted HR* | Crude HR | Adjusted HR* |
| **Hospitalization for infection** |  |  |  |  |  |  |  |
| All infections | 1.0 (Reference) | 0.96 (0.85-1.08) | 1.04 (0.92-1.18) | 0.86 (0.79-0.94) | 0.97 (0.89-1.05) | 1.59 (1.45-1.74) | 1.26 (1.15-1.38) |
| Septicemia | 1.0 (Reference) | 0.87 (0.66-1.15) | 0.96 (0.72-1.27) | 0.82 (0.67-0.99) | 0.94 (0.77-1.14) | 1.59 (1.30-1.94) | 1.23 (1.01-1.51) |
| Lower respiratory tract | 1.0 (Reference) | 0.83 (0.67-1.04) | 1.09 (0.87-1.36) | 0.78 (0.66-0.91) | 0.93 (0.80-1.09) | 1.68 (1.45-1.96) | 1.30 (1.11-1.52) |
| Intra-abdominal | 1.0 (Reference) | 1.17 (0.84-1.62) | 1.17 (0.84-1.62) | 0.79 (0.60-1.03) | 0.82 (0.62-1.07) | 1.42 (1.07-1.90) | 1.25 (0.94-1.67) |
| Reproductive and urinary tract | 1.0 (Reference) | 0.92 (0.75-1.12) | 0.95 (0.77-1.16) | 0.92 (0.80-1.05) | 1.02 (0.89-1.17) | 1.72 (1.49-1.98) | 1.34 (1.16-1.55) |
| Skin and soft tissue | 1.0 (Reference) | 1.00 (0.72-1.38) | 0.91 (0.66-1.27) | 0.84 (0.67-1.07) | 0.93 (0.73-1.17) | 1.11 (0.83-1.49) | 0.91 (0.67-1.22) |
| Osteomyelitis | 1.0 (Reference) | 1.03 (0.46-2.32) | 1.07 (0.47-2.43) | 0.60 (0.30-1.22) | 0.66 (0.32-1.33) | 1.26 (0.62-2.55) | 0.95 (0.47-1.93) |
| Necrotizing fasciitis | 1.0 (Reference) | 0.79 (0.11-5.72) | 0.56 (0.08-4.10) | 0.69 (0.17-2.84) | 0.71 (0.17-2.93) | 2.17 (0.67-6.99) | 1.73 (0.53-5.64) |
| Infectious intestinal diseases | 1.0 (Reference) | 0.41 (0.13-1.28) | 0.45 (0.14-1.40) | 0.85 (0.50-1.46) | 0.90 (0.53-1.55) | 1.90 (1.13-3.20) | 1.58 (0.93-2.66) |
| **Infection-related deaths** | 1.0 (Reference) | 1.00 (0.55-1.81) | 1.60 (0.87-2.94) | 0.59 (0.36-0.98) | 0.86 (0.52-1.42) | 2.07 (1.39-3.08) | 1.40 (0.93-2.09) |

*Adjusted for continuous age, sex, BMI category, smoking (current, non-current), alcohol consumption, education level, diabetes (no, fasting glucose ≤130, 131-200, >200), eGFR category, systemic steroids use >30 days before study entry, and history of hospitalization within 6 months before hospitalization for infection syndrome.

**Abbreviations: ALT, alanine aminotransferase; BMI, body mass index; eGFR, estimated glomerular filtration rate; HR, hazard ratio; NBNC, no HBV or HCV infection; NC-HBV, noncirrhotic with HBV infection; NC-HCV, noncirrhotic with HCV infection;** **UNL, upper normal limit**
