# Supplementary material for: Incidence of hospitalization for infection among patients with hepatitis B or C virus infection without cirrhosis in Taiwan: A cohort study
Source: PLoS Med. 2019 Sep 13;16(9):e1002894. doi: 10.1371/journal.pmed.1002894 (PMC6743759; doi:10.1371/journal.pmed.1002894)
Supplement: S10 Table — (DOCX) [file pmed.1002894.s010.docx]

**S10 Table. The association between different liver disease categories and risk of hospitalization for infection syndrome and infection-related mortality compared with NBNC patients with normal to mildly elevated liver enzyme levels in men (N = 41,005).**

|  | NBNC  ALT normal to 1.5x UNL | NBNC  ALT ≥ 1.5x UNL | | NC-HBV | | NC-HCV | |
| --- | --- | --- | --- | --- | --- | --- | --- |
|  | HR | Crude HR | Adjusted HR* | Crude HR | Adjusted HR* | Crude HR | Adjusted HR* |
| **Hospitalization for infection** |  |  |  |  |  |  |  |
| All infections | 1.0 (Reference) | 0.71 (0.61-0.82) | 1.09 (0.94-1.26) | 0.68 (0.61-0.76) | 0.94 (0.84-1.05) | 1.61 (1.40-1.84) | 1.22 (1.06-1.39) |
| Septicemia | 1.0 (Reference) | 0.48 (0.32-0.70) | 0.83 (0.56-1.24) | 0.55 (0.42-0.72) | 0.85 (0.65-1.11) | 1.64 (1.21-2.23) | 1.22 (0.90-1.66) |
| Lower respiratory tract | 1.0 (Reference) | 0.55 (0.42-0.71) | 1.19 (0.92-1.56) | 0.59 (0.49-0.71) | 0.95 (0.79-1.15) | 1.96 (1.60-2.39) | 1.36 (1.11-1.66) |
| Intra-abdominal | 1.0 (Reference) | 1.24 (0.94-1.65) | 1.27 (0.95-1.69) | 0.71 (0.54-0.93) | 0.77 (0.58-1.02) | 1.31 (0.89-1.94) | 1.15 (0.78-1.71) |
| Reproductive and urinary tract | 1.0 (Reference) | 0.46 (0.32-0.65) | 0.76 (0.53-1.08) | 0.73 (0.60-0.90) | 1.06 (0.86-1.31) | 1.43 (1.07-1.91) | 1.11 (0.83-1.48) |
| Skin and soft tissue | 1.0 (Reference) | 0.82 (0.58-1.16) | 0.94 (0.66-1.34) | 0.70 (0.53-0.93) | 0.88 (0.66-1.16) | 1.28 (0.86-1.90) | 1.00 (0.67-1.49) |
| Osteomyelitis | 1.0 (Reference) | 0.99 (0.44-2.26) | 1.16 (0.50-2.69) | 0.44 (0.18-1.07) | 0.59 (0.24-1.44) | 1.68 (0.68-4.11) | 1.30 (0.53-3.19) |
| Necrotizing fasciitis | 1.0 (Reference) | NA | NA | 0.41 (0.06-3.07) | 0.58 (0.08-4.38) | 4.96 (1.49-16.5) | 3.83 (1.13-13.0) |
| Infectious intestinal diseases | 1.0 (Reference) | 0.76 (0.34-1.73) | 0.98 (0.43-2.25) | 0.68 (0.36-1.29) | 0.84 (0.44-1.59) | 0.79 (0.25-2.49) | 0.64 (0.20-2.02) |
| **Infection-related deaths** | 1.0 (Reference) | 0.73 (0.37-1.42) | 2.18 (1.11-4.30) | 0.38 (0.19-0.73) | 0.78 (0.40-1.51) | 1.95 (1.09-3.49) | 1.33 (0.74-2.38) |

*Adjusted for continuous age, BMI category, smoking (current, non-current), alcohol consumption, education level, diabetes (no, fasting glucose ≤130, 131-200, >200), eGFR category, systemic steroids use >30 days before study entry, and history of hospitalization within 6 months before hospitalization for infection syndrome.

**Abbreviations: ALT, alanine aminotransferase; BMI, body mass index; eGFR, estimated glomerular filtration rate; HR, hazard ratio; NA, not applicable; NBNC, no HBV or HCV infection; NC-HBV, noncirrhotic with HBV infection; NC-HCV, noncirrhotic with HCV infection; UNL, upper normal limit**
