# Supplementary material for: Incidence of hospitalization for infection among patients with hepatitis B or C virus infection without cirrhosis in Taiwan: A cohort study
Source: PLoS Med. 2019 Sep 13;16(9):e1002894. doi: 10.1371/journal.pmed.1002894 (PMC6743759; doi:10.1371/journal.pmed.1002894)
Supplement: S11 Table — (DOCX) [file pmed.1002894.s011.docx]

**S11 Table. The association between different liver disease categories and risk of hospitalization for infection syndrome and infection-related mortality compared with NBNC patients with normal to mildly elevated liver enzyme levels in women (N = 74,331).**

|  | NBNC  ALT normal to 1.5x UNL | NBNC  ALT ≥ 1.5x UNL | | NC-HBV | | NC-HCV | |
| --- | --- | --- | --- | --- | --- | --- | --- |
|  | HR | Crude HR | Adjusted HR* | Crude HR | Adjusted HR* | Crude HR | Adjusted HR* |
| **Hospitalization for infection** |  |  |  |  |  |  |  |
| All infections | 1.0 (Reference) | 1.30 (1.13-1.48) | 1.06 (0.93-1.22) | 0.89 (0.81-0.97) | 0.95 (0.87-1.04) | 1.83 (1.65-2.04) | 1.21 (1.09-1.35) |
| Septicemia | 1.0 (Reference) | 1.39 (1.01-1.93) | 1.08 (0.78-1.50) | 0.83 (0.66-1.04) | 0.92 (0.73-1.15) | 2.21 (1.73-2.82) | 1.27 (0.99-1.62) |
| Lower respiratory tract | 1.0 (Reference) | 0.96 (0.69-1.34) | 0.87 (0.62-1.22) | 0.73 (0.59-0.90) | 0.84 (0.68-1.03) | 1.99 (1.59-2.48) | 1.12 (0.90-1.40) |
| Intra-abdominal | 1.0 (Reference) | 1.30 (0.88-1.92) | 1.17 (0.79-1.74) | 0.82 (0.62-1.07) | 0.84 (0.64-1.11) | 1.61 (1.16-2.23) | 1.25 (0.89-1.74) |
| Reproductive and urinary tract | 1.0 (Reference) | 1.40 (1.17-1.68) | 1.11 (0.92-1.34) | 0.95 (0.84-1.08) | 1.00 (0.88-1.13) | 1.91 (1.65-2.22) | 1.34 (1.15-1.55) |
| Skin and soft tissue | 1.0 (Reference) | 1.22 (0.81-1.82) | 0.85 (0.57-1.28) | 0.95 (0.74-1.23) | 1.06 (0.82-1.37) | 1.32 (0.92-1.90) | 0.85 (0.59-1.23) |
| Osteomyelitis | 1.0 (Reference) | 0.75 (0.18-3.02) | 0.62 (0.15-2.52) | 0.65 (0.29-1.47) | 0.73 (0.32-1.65) | 1.29 (0.48-3.50) | 0.69 (0.25-1.87) |
| Necrotizing fasciitis | 1.0 (Reference) | 1.86 (0.25-13.8) | 0.64 (0.08-4.92) | 1.15 (0.27-4.87) | 1.24 (0.29-5.29) | NA | NA |
| Infectious intestinal diseases | 1.0 (Reference) | NA | NA | 0.97 (0.13-7.52) | 0.92 (0.12-7.20) | NA | NA |
| **Infection-related deaths** | 1.0 (Reference) | 2.27 (0.30-16.9) | 2.67 (0.35-20.2) | 1.38 (0.32-5.92) | 1.31 (0.30-5.66) | 3.99 (0.93-17.1) | 2.10 (0.46-9.56) |

*Adjusted for continuous age, BMI category, smoking (current, non-current), alcohol consumption, education level, diabetes (no, fasting glucose ≤130, 131-200, >200), eGFR category, systemic steroids use >30 days before study entry, and history of hospitalization within 6 months before hospitalization for infection syndrome.

**Abbreviations: ALT, alanine aminotransferase; BMI, body mass index; eGFR, estimated glomerular filtration rate; HR, hazard ratio; NA, not applicable; NBNC, no HBV or HCV infection; NC-HBV, noncirrhotic with HBV infection; NC-HCV, noncirrhotic with HCV infection;** **UNL, upper normal limit**
