# Supplementary material for: Incidence of hospitalization for infection among patients with hepatitis B or C virus infection without cirrhosis in Taiwan: A cohort study
Source: PLoS Med. 2019 Sep 13;16(9):e1002894. doi: 10.1371/journal.pmed.1002894 (PMC6743759; doi:10.1371/journal.pmed.1002894)
Supplement: S12 Table — (DOCX) [file pmed.1002894.s012.docx]

**S12 Table.** **Baseline demographics, comorbidities, medication use, and resource utilization, measured within 1 year before the index date among HCV patients who received and those who did not receive antiviral therapy before and after PS matching.**

|  | Original study cohort before PS matching  (N=124,624) | | | 1:5 variable-ratio PS-matched cohort  (N=68,723) | | | 1:5 variable-ratio hd-PS-matched cohort  (N=59,437) | | |
| --- | --- | --- | --- | --- | --- | --- | --- | --- | --- |
|  | HCV patients who received antiviral therapy  (N=20,264) | HCV patients who did not receive antiviral therapy  (N=104,360) | Standardized difference | HCV patients who received antiviral therapy  (N=16,558) | HCV patients who did not receive antiviral therapy  (N=52,165) | Standardized difference | HCV patients who received antiviral therapy  (N=15,807) | HCV patients who did not receive antiviral therapy  (N=43,630) | Standardized difference |
| **Demographics** |  |  |  |  |  |  |  |  |  |
| Age at hepatitis C diagnosis in years, mean (SD) | 52.12 (11.86) | 55.62 (16.27) | -0.245 | 52.55 (11.84) | 52.94 (15.94) | -0.027 | 52.75 (11.80) | 52.75 (15.35) | 0.000 |
| Men, % | 55.37 | 46.11 | 0.186 | 52.75 | 52.67 | 0.002 | 52.08 | 51.64 | 0.009 |
| Interval between the date of first hepatitis C visit and the start of follow-up in days, mean (SD) | 1395.65 (1128.36) | 1158.76 (875.78) | 0.235 | 1329.37 (1109.78) | 1296.32 (891.83) | 0.033 | 1259.27 (1081.14) | 1238.32 (905.90) | 0.021 |
| Moderate or severe liver disease | 0.15 | 0.09 | 0.016 | 0.11 | 0.11 | 0.002 | 0.15 | 0.16 | -0.005 |
| **Comorbidities, %** |  |  |  |  |  |  |  |  |  |
| Diabetes | 18.10 | 17.43 | 0.018 | 17.81 | 18.49 | -0.018 | 17.97 | 17.29 | 0.018 |
| Hypertension | 28.72 | 32.45 | -0.081 | 29.22 | 29.75 | -0.012 | 29.59 | 29.34 | 0.006 |
| Ischemic heart disease | 6.87 | 9.23 | -0.087 | 7.14 | 7.60 | -0.018 | 7.34 | 7.65 | -0.012 |
| Myocardial infarction | 0.30 | 0.61 | -0.046 | 0.32 | 0.28 | 0.007 | 0.34 | 0.33 | 0.002 |
| Cardiac dysrhythmia/atrial fibrillation | 3.84 | 5.31 | -0.070 | 4.01 | 4.07 | -0.003 | 4.13 | 4.04 | 0.005 |
| Congestive heart failure | 1.71 | 3.92 | -0.134 | 1.82 | 2.00 | -0.013 | 1.92 | 2.00 | -0.005 |
| Stroke | 1.74 | 4.46 | -0.158 | 1.83 | 1.86 | -0.002 | 1.87 | 1.89 | -0.002 |
| Peripheral vascular disease | 0.91 | 1.21 | -0.029 | 0.95 | 1.00 | -0.006 | 1.00 | 0.95 | 0.005 |
| Disorders of lipid metabolism | 18.09 | 17.66 | 0.011 | 18.38 | 18.67 | -0.007 | 18.87 | 18.81 | 0.001 |
| Chronic lung disease | 8.52 | 12.47 | -0.129 | 8.85 | 9.64 | -0.027 | 9.10 | 8.96 | 0.005 |
| Chronic kidney disease | 1.08 | 1.98 | -0.074 | 1.17 | 1.27 | -0.009 | 1.22 | 1.28 | -0.005 |
| Dementia | 0.27 | 2.57 | -0.195 | 0.31 | 0.49 | -0.029 | 0.34 | 0.39 | -0.009 |
| Peptic ulcer disease | 35.46 | 29.77 | 0.122 | 34.34 | 35.73 | -0.029 | 35.21 | 35.60 | -0.008 |
| Charlson comorbidity score (excluding liver disease) | 0.62 (0.89) | 0.73 (1.15) | -0.109 | 0.62 (0.90) | 0.65 (0.97) | -0.033 | 0.64 (0.91) | 0.63 (0.93) | 0.014 |
| **Medication use (%)** |  |  |  |  |  |  |  |  |  |
| Proton pump inhibitor or H2-receptor blocker use | 38.67 | 33.93 | 0.099 | 37.63 | 38.75 | -0.023 | 38.20 | 38.84 | -0.013 |
| Systemic steroid use > 30 days | 2.15 | 3.44 | -0.079 | 2.21 | 2.31 | -0.007 | 2.25 | 2.17 | 0.005 |
| Antibiotics | 51.21 | 52.15 | -0.019 | 51.28 | 52.11 | -0.017 | 51.93 | 51.91 | 0.000 |
| **Resource utilization within 1 year before index date (%)** |  |  |  |  |  |  |  |  |  |
| History of hospitalization due to infection related episodes ^*^ | 2.12 | 6.57 | -0.219 | 2.29 | 2.77 | -0.030 | 2.45 | 2.73 | -0.018 |
| History of hospitalization due to gastrointestinal bleeding | 2.09 | 2.12 | -0.002 | 1.65 | 1.85 | -0.015 | 1.88 | 1.96 | -0.006 |
| History of hospitalization due to liver disease related episodes ^†^ | 18.74 | 5.54 | 0.413 | 9.49 | 10.38 | -0.029 | 9.45 | 10.39 | -0.032 |
| Number of hospitalization, mean (SD) | 0.30 (0.62) | 0.28 (0.86) | 0.029 | 0.21 (0.55) | 0.23 (0.61) | -0.034 | 0.21 (0.55) | 0.23 (0.59) | -0.029 |
| Number of outpatient visits, mean (SD) | 26.67 (17.50) | 21.77 (19.78) | 0.262 | 25.71 (16.50) | 26.70 (24.32) | -0.048 | 25.93 (16.62) | 26.26 (22.92) | -0.017 |
| Number of outpatient visits due to infection related episodes ^*^, mean (SD) | 0.97 (2.47) | 1.11 (2.82) | -0.052 | 0.99 (2.53) | 1.03 (2.52) | -0.015 | 1.01 (2.56) | 1.02 (2.49) | -0.004 |

**Abbreviations: HCV, hepatitis C virus; hd-PS, high-dimensional propensity score; PS, propensity score; SD, standard deviation.**

* Infection related episodes included septicemia, lower respiratory tract infection, intra-abdominal infection, reproductive urinary tract infection, skin and soft tissue infection, osteomyelitis, necrotizing fasciitis, and central nerve infection.

† Liver disease related episodes included acute or chronic hepatitis with or without hepatic coma

C-statistics for propensity score model = 0.808

C-statistics for hd-propensity score model = 0.875
