# Supplementary material for: Incidence of hospitalization for infection among patients with hepatitis B or C virus infection without cirrhosis in Taiwan: A cohort study
Source: PLoS Med. 2019 Sep 13;16(9):e1002894. doi: 10.1371/journal.pmed.1002894 (PMC6743759; doi:10.1371/journal.pmed.1002894)
Supplement: S13 Table — (DOCX) [file pmed.1002894.s013.docx]

**S13 Table.** **Follow-up duration, number of incident cases, and crude incidence of hospitalization for infection syndrome and infection-related mortality among HCV patients who received and those who did not receive antiviral therapy before and after PS and hd-PS matching.**

|  | Original study cohort before PS matching  (N=124,624) | | 1:5 variable-ratio PS-matched cohort  (N=68,723) | | 1:5 variable-ratio hd-PS-matched cohort  (N=59,437) | |
| --- | --- | --- | --- | --- | --- | --- |
|  | HCV patients who received antiviral therapy  (N=20,264) | HCV patients who did not receive antiviral therapy (N=104,360) | HCV patients who received antiviral therapy  (N=16,558) | HCV patients who did not receive antiviral therapy (N=52,165) | HCV patients who received antiviral therapy  (N=15,807) | HCV patients who did not receive antiviral therapy (N=43,630) |
| **N=** | 20,264 | 104,360 | 16,558 | 52,165 | 15,807 | 43,630 |
| **Follow-up duration** | 32,889,211 | 162,528,601 | 25,614,085 | 88,709,407 | 24,614,095 | 73,586,890 |
| **Total person-years** | 90,046 | 444,979 | 70,128 | 242,873 | 67,390 | 201,470 |
| **Median follow-up years (interquartile range)** | 4.16 (3.38) | 4.07 (3.77) | 4.01 (3.20) | 4.50 (3.65) | 4.06 (3.19) | 4.38 (3.79) |
| **Hospitalization for infection** |  |  |  |  |  |  |
| **All infections** |  |  |  |  |  |  |
| Number of incidence cases | 1,339 | 11,566 | 1,084 | 4,685 | 1,064 | 3,640 |
| Crude incidence rate^†^ | 14.87 (14.09~15.69) | 25.99 (25.52~26.47) | 15.01 (13.73~16.41) | 19.29 (18.75~19.85) | 15.38 (14.14~16.73) | 18.07 (17.49~18.66) |
|  |  |  |  |  |  |  |
| **Septicemia** |  |  |  |  |  |  |
| Number of incidence cases | 259 | 2,845 | 213 | 1,009 | 217 | 762 |
| Crude incidence rate^†^ | 2.88 (2.55~3.25) | 6.39 (6.16~6.63) | 3.06 (2.51~3.73) | 4.15 (3.91~4.42) | 3.15 (2.61~3.79) | 3.78 (3.52~4.06) |
|  |  |  |  |  |  |  |
| **Lower respiratory tract** |  |  |  |  |  |  |
| Number of incidence cases | 376 | 4,959 | 293 | 1,725 | 284 | 1,256 |
| Crude incidence rate^†^ | 4.18 (3.77~4.62) | 11.14 (10.84~11.46) | 4.16 (3.51~4.92) | 7.10 (6.78~7.45) | 4.09 (3.47~4.81) | 6.23 (5.90~6.59) |
|  |  |  |  |  |  |  |
| **Intra-abdominal** |  |  |  |  |  |  |
| Number of incidence cases | 174 | 977 | 136 | 486 | 130 | 409 |
| Crude incidence rate^†^ | 1.93 (1.67~2.24) | 2.20 (2.06~2.34) | 1.79 (1.38~2.31) | 2.00 (1.83~2.19) | 1.75 (1.36~2.25) | 2.03 (1.84~2.24) |
|  |  |  |  |  |  |  |
| **Reproductive and urinary tract** |  |  |  |  |  |  |
| Number of incidence cases | 458 | 4,472 | 394 | 1,665 | 389 | 1,303 |
| Crude incidence rate^†^ | 5.09 (4.64~5.57) | 10.05 (9.76~10.35) | 5.26 (4.52~6.11) | 6.86 (6.53~7.19) | 5.38 (4.67~6.21) | 6.47 (6.13~6.83) |
|  |  |  |  |  |  |  |
| **Skin and soft tissue** |  |  |  |  |  |  |
| Number of incidence cases | 226 | 1,532 | 176 | 729 | 175 | 548 |
| Crude incidence rate^†^ | 2.51 (2.20~2.86) | 3.44 (3.27~3.62) | 2.54 (2.05~3.16) | 3.00 (2.79~3.23) | 2.75 (2.26~3.36) | 2.72 (2.50~2.96) |
|  |  |  |  |  |  |  |
| **Osteomyelitis** |  |  |  |  |  |  |
| Number of incidence cases | 32 | 179 | 30 | 85 | 30 | 64 |
| Crude incidence rate^†^ | 0.36 (0.25~0.50) | 0.40 (0.35~0.47) | 0.43 (0.26~0.73) | 0.35 (0.28~0.43) | 0.47 (0.29~0.77) | 0.32 (0.25~0.41) |
|  |  |  |  |  |  |  |
| **Necrotizing fasciitis** |  |  |  |  |  |  |
| Number of incidence cases | 12 | 120 | 10 | 61 | 6 | 46 |
| Crude incidence rate^†^ | 0.13 (0.08~0.23) | 0.27 (0.23~0.32) | 0.23 (0.11~0.47) | 0.25 (0.20~0.32) | 0.13 (0.05~0.32) | 0.23 (0.17~0.30) |
| **Infectious intestinal diseases** |  |  |  |  |  |  |
| Number of incidence cases | 74 | 344 | 59 | 177 | 60 | 138 |
| Crude incidence rate^†^ | 0.82 (0.65~1.03) | 0.77 (0.70~0.86) | 0.84 (0.58~1.22) | 0.73 (0.63~0.84) | 0.97 (0.70~1.36) | 0.68 (0.58~0.81) |
| **Infection-related deaths** |  |  |  |  |  |  |
| Number of cases | 12 | 1,548 | 8 | 335 | 10 | 241 |
| Crude mortality rate^†^ | 0.13 (0.07~0.22) | 3.31 (3.15~3.48) | 0.13 (0.05~0.33) | 1.32 (1.18~1.47) | 0.18 (0.08~0.39) | 1.14 (1.01~1.30) |

**Abbreviations: HCV, hepatitis C virus; hd-PS, high-dimensional propensity score; PS, propensity score; SD, standard deviation.**

† Crude incidence rate or mortality rate per 1,000 person-years.
