# Supplementary material for: Incidence of hospitalization for infection among patients with hepatitis B or C virus infection without cirrhosis in Taiwan: A cohort study
Source: PLoS Med. 2019 Sep 13;16(9):e1002894. doi: 10.1371/journal.pmed.1002894 (PMC6743759; doi:10.1371/journal.pmed.1002894)
Supplement: S14 Table — (DOCX) [file pmed.1002894.s014.docx]

**S14 Table.** **Baseline demographics, comorbidities, medication use, and resource utilization, measured within 1 year before the index date among HBV patients who received and those who did not receive antiviral therapy before and after PS and hd-PS matching.**

|  | Original study cohort before PS matching  (N=686,789) | | | 1:5 variable-ratio PS-matched cohort  (N=130,153) | | | 1:5 variable-ratio hd-PS-matched cohort  (N=124,970) | | |
| --- | --- | --- | --- | --- | --- | --- | --- | --- | --- |
|  | HBV patients who received antiviral therapy  (N=25,453) | HBV patients who did not receive antiviral therapy  (N=661,336) | Standardized difference | HBV patients who received antiviral therapy  (N=24,211) | HBV patients who did not receive antiviral therapy  (N=105,942) | Standardized difference | HBV patients who received antiviral therapy  (N=24,069) | HBV patients who did not receive antiviral therapy  (N=100,901) | Standardized difference |
| **Demographics** |  |  |  |  |  |  |  |  |  |
| Age at hepatitis B diagnosis in years, mean (SD) | 42.90 (12.78) | 44.25 (13.80) | -0.102 | 43.03 (12.75) | 43.49 (13.45) | -0.035 | 43.04 (12.74) | 43.12 (13.41) | -0.006 |
| Men, % | 72.31 | 55.42 | 0.357 | 71.37 | 71.56 | -0.004 | 71.34 | 71.07 | 0.006 |
| Moderate or severe liver disease | 2.52 | 0.04 | 0.222 | 0.97 | 0.59 | 0.042 | 0.85 | 0.58 | 0.032 |
| **Comorbidities, %** |  |  |  |  |  |  |  |  |  |
| Diabetes | 11.02 | 8.53 | 0.084 | 10.97 | 11.89 | -0.029 | 10.96 | 11.31 | -0.011 |
| Hypertension | 16.16 | 15.90 | 0.007 | 16.31 | 17.30 | -0.027 | 16.30 | 16.60 | -0.008 |
| Ischemic heart disease | 4.34 | 4.10 | 0.012 | 4.35 | 4.79 | -0.021 | 4.39 | 4.47 | -0.004 |
| Myocardial infarction | 0.41 | 0.22 | 0.034 | 0.40 | 0.41 | -0.001 | 0.39 | 0.43 | -0.006 |
| Cardiac dysrhythmia/atrial fibrillation | 2.60 | 2.38 | 0.014 | 2.59 | 2.62 | -0.002 | 2.62 | 2.74 | -0.007 |
| Congestive heart failure | 1.33 | 1.03 | 0.028 | 1.27 | 1.37 | -0.009 | 1.28 | 1.29 | -0.001 |
| Stroke | 1.42 | 1.22 | 0.017 | 1.40 | 1.51 | -0.009 | 1.42 | 1.52 | -0.009 |
| Peripheral vascular disease | 0.38 | 0.46 | -0.013 | 0.40 | 0.49 | -0.014 | 0.38 | 0.47 | -0.013 |
| Disorders of lipid metabolism | 17.91 | 14.64 | 0.089 | 18.13 | 19.20 | -0.027 | 18.17 | 18.52 | -0.009 |
| Chronic lung disease | 6.58 | 6.44 | 0.005 | 6.62 | 6.57 | 0.002 | 6.58 | 6.62 | -0.002 |
| Chronic kidney disease | 0.80 | 0.65 | 0.018 | 0.80 | 0.84 | -0.005 | 0.82 | 0.89 | -0.008 |
| Dementia | 0.27 | 0.40 | -0.023 | 0.27 | 0.35 | -0.014 | 0.27 | 0.32 | -0.011 |
| Peptic ulcer disease | 33.07 | 21.14 | 0.271 | 32.05 | 34.17 | -0.045 | 32.19 | 32.56 | -0.008 |
| Charlson comorbidity score (excluding liver disease) | 0.47 (0.81) | 0.33 (0.72) | 0.172 | 0.46 (0.80) | 0.48 (0.84) | -0.032 | 0.46 (0.81) | 0.47 (0.84) | -0.015 |
| **Medication use (%)** |  |  |  |  |  |  |  |  |  |
| Proton pump inhibitor or H2-receptor blocker use | 35.01 | 24.53 | 0.231 | 34.15 | 35.73 | -0.033 | 34.28 | 35.19 | -0.019 |
| Systemic steroid use > 30 days | 1.86 | 1.23 | 0.051 | 1.83 | 2.06 | -0.016 | 1.82 | 1.89 | -0.005 |
| Antibiotics | 50.24 | 47.84 | 0.048 | 50.40 | 53.22 | -0.057 | 50.34 | 51.89 | -0.031 |
| **Resource utilization within 1 year before index date (%)** |  |  |  |  |  |  |  |  |  |
| History of hospitalization due to infection related episodes ^*^ | 3.92 | 1.84 | 0.125 | 3.79 | 4.10 | -0.016 | 3.78 | 4.00 | -0.011 |
| History of hospitalization due to gastrointestinal bleeding | 4.03 | 0.55 | 0.234 | 3.24 | 3.00 | 0.014 | 3.39 | 3.22 | 0.010 |
| History of hospitalization due to liver disease related episodes ^†^ | 24.41 | 2.05 | 0.699 | 20.57 | 18.63 | 0.049 | 20.09 | 18.52 | 0.040 |
| Number of hospitalization, mean (SD) | 0.37 (0.70) | 0.11 (0.44) | 0.452 | 0.33 (0.68) | 0.32 (0.69) | 0.019 | 0.33 (0.68) | 0.32 (0.73) | 0.014 |
| Number of outpatient visits, mean (SD) | 20.36 (14.84) | 14.09 (13.86) | 0.437 | 20.28 (14.42) | 22.46 (21.16) | -0.120 | 20.27 (14.44) | 21.81 (20.75) | -0.086 |
| Number of outpatient visits due to infection related episodes ^*^, mean (SD) | 0.74 (2.03) | 0.78 (2.07) | -0.019 | 0.75 (2.05) | 0.79 (2.01) | -0.020 | 0.75 (2.06) | 0.76 (1.93) | -0.005 |

**Abbreviations: HBV, hepatitis B virus; hd-PS, high-dimensional propensity score; PS, propensity score; SD, standard deviation.**

PS, propensity score; SD, standard deviation; hd-PS, high-dimensional propensity score.

* Infection related episodes included septicemia, lower respiratory tract infection, intra-abdominal infection, reproductive urinary tract infection, skin and soft tissue infection, osteomyelitis, necrotizing fasciitis, and central nerve infection.

† Liver disease related episodes included acute or chronic hepatitis with or without hepatic coma

C-statistics for propensity score model = 0.839

C-statistics for hd-propensity score model = 0.891
