# Supplementary material for: Incidence of hospitalization for infection among patients with hepatitis B or C virus infection without cirrhosis in Taiwan: A cohort study
Source: PLoS Med. 2019 Sep 13;16(9):e1002894. doi: 10.1371/journal.pmed.1002894 (PMC6743759; doi:10.1371/journal.pmed.1002894)
Supplement: S15 Table — (DOCX) [file pmed.1002894.s015.docx]

**S15 Table.** **Baseline demographics, comorbidities, medication use, and resource utilization, measured within 1 year before the index date among HBV patients who received and those who did not receive antiviral therapy before and after PS and hd-PS matching.**

|  | Original study cohort before PS matching  (N=686,789) | | 1:5 variable-ratio PS-matched cohort  (N=130,153) | | 1:5 variable-ratio hd-PS-matched cohort  (N=124,970) | |
| --- | --- | --- | --- | --- | --- | --- |
|  | HBV patients who received antiviral therapy  (N=25,453) | HBV patients who did not receive antiviral therapy (N=661,336) | HBV patients who received antiviral therapy  (N=24,211) | HBV patients who did not receive antiviral therapy (N=105,942) | HBV patients who received antiviral therapy  (N=24,069) | HBV patients who did not receive antiviral therapy (N=100,901) |
| **N=** | 25,453 | 661,336 | 24,211 | 105,942 | 24,069 | 100,901 |
| **Follow-up duration** | 44,137,765 | 1,129,216,338 | 41,626,006 | 201,180,613 | 41,323,217 | 194,607,591 |
| **Total person-years** | 120,843 | 3,091,626 | 113,966 | 550,802 | 113,137 | 532,807 |
| **Median follow-up years (interquartile range)** | 4.43 (3.88) | 4.38 (4.04) | 4.38 (3.73) | 4.88 (4.90) | 4.38 (3.71) | 4.97 (5.31) |
| **Hospitalization for infection** |  |  |  |  |  |  |
| **All infections** |  |  |  |  |  |  |
| Number of incidence cases | 1,052 | 28,095 | 976 | 5,392 | 975 | 4,769 |
| Crude incidence rate^†^ | 8.71 (8.20~9.25) | 9.09 (8.98~9.19) | 8.56 (8.04~9.12) | 10.48(9.93~11.06) | 8.62 (8.09~9.18) | 10.08 (9.54~10.66) |
|  |  |  |  |  |  |  |
| **Septicemia** |  |  |  |  |  |  |
| Number of incidence cases | 188 | 4,366 | 171 | 753 | 170 | 735 |
| Crude incidence rate^†^ | 1.56 (1.35~1.79) | 1.41 (1.37~1.45) | 1.50 (1.29~1.74) | 1.50 (1.30~1.73) | 1.50 (1.29~1.75) | 1.51 (1.31~1.74) |
|  |  |  |  |  |  |  |
| **Lower respiratory tract** |  |  |  |  |  |  |
| Number of incidence cases | 331 | 9,069 | 309 | 1,746 | 306 | 1,528 |
| Crude incidence rate^†^ | 2.74 (2.46~3.05) | 2.93 (2.87~2.99) | 2.71 (2.43~3.03) | 3.33 (3.03~3.67) | 2.70 (2.42~3.03) | 3.34 (3.04~3.68) |
|  |  |  |  |  |  |  |
| **Intra-abdominal** |  |  |  |  |  |  |
| Number of incidence cases | 218 | 4,945 | 207 | 976 | 202 | 908 |
| Crude incidence rate^†^ | 1.80 (1.58~2.06) | 1.60 (1.56~1.64) | 1.82 (1.59~2.08) | 1.90 (1.67~2.16) | 1.79 (1.56~2.05) | 1.65 (1.44~1.89) |
|  |  |  |  |  |  |  |
| **Reproductive and urinary tract** |  |  |  |  |  |  |
| Number of incidence cases | 274 | 8,840 | 257 | 1,406 | 259 | 1,278 |
| Crude incidence rate^†^ | 2.27 (2.01~2.55) | 2.86 (2.80~2.92) | 2.26 (2.00~2.55) | 2.47 (2.21~2.76) | 2.29 (2.03~2.59) | 2.71 (2.44~3.02) |
|  |  |  |  |  |  |  |
| **Skin and soft tissue** |  |  |  |  |  |  |
| Number of incidence cases | 142 | 4,474 | 127 | 996 | 133 | 802 |
| Crude incidence rate^†^ | 1.18 (1.00~1.39) | 1.45 (1.41~1.49) | 1.11 (0.94~1.33) | 2.06 (1.82~2.33) | 1.18 (0.99~1.39) | 1.84 (1.62~2.10) |
|  |  |  |  |  |  |  |
| **Osteomyelitis** |  |  |  |  |  |  |
| Number of incidence cases | 12 | 357 | 10 | 78 | 11 | 53 |
| Crude incidence rate^†^ | 0.10 (0.06~0.17) | 0.12 (0.10~0.13) | 0.09 (0.05~0.16) | 0.17 (0.11~0.26) | 0.10 (0.05~0.18) | 0.16 (0.10~0.25) |
|  |  |  |  |  |  |  |
| **Necrotizing fasciitis** |  |  |  |  |  |  |
| Number of incidence cases | 8 | 224 | 7 | 49 | 7 | 47 |
| Crude incidence rate^†^ | 0.07 (0.03~0.13) | 0.07 (0.06~0.08) | 0.06 (0.03~0.13) | 0.10 (0.06~0.18) | 0.06 (0.03~0.13) | 0.09 (0.05~0.16) |
| **Infectious intestinal diseases** |  |  |  |  |  |  |
| Number of incidence cases | 60 | 1,356 | 58 | 290 | 56 | 254 |
| Crude incidence rate^†^ | 0.50 (0.39~0.64) | 0.44 (0.42~0.46) | 0.51 (0.39~0.66) | 0.61 (0.49~0.77) | 0.49 (0.38~0.64) | 0.41 (0.31~0.54) |
| **Infection-related deaths** |  |  |  |  |  |  |
| Number of cases | 46 | 1,225 | 44 | 247 | 44 | 251 |
| Crude mortality rate^†^ | 0.37 (0.28~0.50) | 0.39 (0.37~0.41) | 0.38 (0.28~0.51) | 0.49 (0.38~0.62) | 0.38 (0.28~0.51) | 0.66 (0.54~0.82) |

**Abbreviations: HBV, hepatitis B virus; hd-PS, high-dimensional propensity score; PS, propensity score**

† Crude incidence rate or mortality rate per 1,000 person-years.
