# Supplementary material for: Incidence of hospitalization for infection among patients with hepatitis B or C virus infection without cirrhosis in Taiwan: A cohort study
Source: PLoS Med. 2019 Sep 13;16(9):e1002894. doi: 10.1371/journal.pmed.1002894 (PMC6743759; doi:10.1371/journal.pmed.1002894)
Supplement: S16 Table — (DOCX) [file pmed.1002894.s016.docx]

**S16 Table.** **Risk of hospitalization for infection syndrome and infection-related mortality comparing HBV patients who received antiviral therapy to those who did not receive antiviral therapy.**

|  | HBV patients who did not receive antiviral therapy | HBV patients who received antiviral therapy | | |
| --- | --- | --- | --- | --- |
|  | HR | HR before PS matching (95% CI) | HR after PS matching (95% CI) | HR after hd-PS matching (95% CI) |
| **Hospitalization for infection** |  |  |  |  |
| All infections | Reference | 0.96 (0.90-1.02) | 0.80 (0.73-0.87) | 0.83 (0.76-0.90) |
| Septicemia | Reference | 1.10 (0.95-1.28) | 0.97 (0.79-1.19) | 0.97 (0.78-1.19) |
| Lower respiratory tract | Reference | 0.94 (0.84-1.04) | 0.80 (0.69-0.92) | 0.79 (0.68-0.91) |
| Intra-abdominal | Reference | 1.13 (0.99-1.29) | 0.94 (0.78-1.13) | 1.07 (0.88-1.30) |
| Reproductive and urinary tract | Reference | 0.79 (0.70-0.90) | 0.89 (0.75-1.05) | 0.81 (0.69-0.95) |
| Skin and soft tissue | Reference | 0.81 (0.69-0.96) | 0.53 (0.43-0.65) | 0.63 (0.51-0.78) |
| Osteomyelitis | Reference | 0.86 (0.48-1.53) | 0.49 (0.23-1.05) | 0.56 (0.27-1.18) |
| Necrotizing fasciitis | Reference | 0.91 (0.45-1.85) | 0.56 (0.22-1.41) | 0.65 (0.25-1.67) |
| Infectious intestinal diseases | Reference | 1.13 (0.87-1.47) | 0.81 (0.58-1.15) | 1.19 (0.81-1.74) |
| **Infection-related deaths** | Reference | 0.96 (0.72-1.29) | 0.73 (0.50-1.08) | 0.54 (0.38-0.78) |

**Abbreviations: CI, confidence interval; HBV, hepatitis B virus; hd-PS, high-dimensional propensity score; HR, hazard ratio; PS, propensity score**
