# Supplementary material for: Incidence of hospitalization for infection among patients with hepatitis B or C virus infection without cirrhosis in Taiwan: A cohort study
Source: PLoS Med. 2019 Sep 13;16(9):e1002894. doi: 10.1371/journal.pmed.1002894 (PMC6743759; doi:10.1371/journal.pmed.1002894)
Supplement: S1 Text — (DOCX) [file pmed.1002894.s017.docx]

**Increased incidence of hospitalization for infection among non-cirrhotic patients with hepatitis C virus infection: a prospective cohort study**

**Study objective:**

To examine the risks of a wide range of clinically important infectious diseases and infection-related mortality among patients infected by HCV using a cohort study design

**Specific research questions:**

1. Do patients infected by HCV have significantly increased risks of infection, as compared with patients with and without other liver diseases?
2. Is there any particular site of infection that HCV patients may have?
3. Does the risk of infection associated with HCV differ by patient characteristics, such as the severity of liver disease or amount of alcohol consumption?

**Database and study period:**

1. New Taipei City Health Screening Database (03/05/2005-07/27/2008)

2. Taiwan National Health Insurance Research Database (01/01/2004-12/31/2015)

3. National Death Registry (03/05/2005-12/31/2015)

**Study population**

**Inclusion criteria**

Adults who participated in New Taipei City Health Screening during 2005 to 2008.

**Exclusion criteria**

1) younger than 20 years old

2) did not have baseline measurement of viral hepatitis marker, liver function test, serum creatinine, BMI, and fasting glucose level

3) did not have complete information about cigarette smoking, alcohol consumption, and education level

4) did not have any inpatient or outpatients records in the National Health Insurance Database.

5) with diagnosis of liver cirrhosis before and after study beginning. Information about liver cirrhosis will be obtained from the National Health Insurance Database.

**Exposure ascertainment**

Participants will be classified into the following four liver disease categories according to their liver function and status of viral hepatitis markers

1. No HBV or HCV infection with mild ALT level normal ~ 1.5x UNL
2. No HBV or HCV infection but with ALT ≥ 1.5x UNL
3. Non-cirrhotic hepatitis B (positive for hepatitis B surface antigen)
4. Non-cirrhotic hepatitis C (positive for anti-hepatitis C antibody)
5. Non-cirrhotic HBV/HCV coinfection

**Other covariates definition and ascertainment**

Information associated with increased susceptibility to clinically significant infectious diseases, including diabetes, renal function, body mass index (BMI), education level, cigarette smoking, alcohol consumption, systemic steroids use, and hospitalization history will be collected.

- Diabetes: if any of the following criteria:

1) fasting plasma glucose (FPG) over 126 mg/dL; or

2) prescription of any hypoglycemic agent (verified from the health insurance claims database) for more than 28 days in the previous year before the baseline survey.

Participants will be classified into the following categories accordingly: no diabetes, diabetes with fasting sugar ≤130, fasting sugar between131-200, and >200 mg/dl.

- Renal function: defined by estimated glomerular filtration rate (eGFR) using the CKD-EPI study equation.

Participants will be classified into the following categories according to their eGFR level and whether they received dialysis therapy: ≥90, 60-89, and <59 mL/min/1.73 m^2^ or receiving dialysis therapy (Information about hemodialysis or peritoneal dialysis will be obtained from the National Health Insurance Database).

- Body mass index will be calculated by dividing self-reported weight (in kilogram) by square of height (in meter).

BMI will be categorized following the WHO classification: underweight (<18.5 kg/m^2^), normal (≥18.5 kg/m^2^ and <25 kg/m^2^), overweight (≥25 kg/m^2^ and <30 kg/m^2^), and obese (≥30 kg/m^2^).

The following variables will be obtained from the questionnaire at cohort entry:

- Age (continuous)
- Sex (men, women)
- Level of education (illiterate, literate but not attending elementary school, elementary school, junior high school, high school, college, graduate school)
- Smoking (current, non-current)
- Alcohol consumption (never, quitted, regular, sometimes)
- Information about comorbid diseases, systemic steroids use >30 days in the year prior to study entry, and history of hospitalization within 6 months before hospitalization for infection syndrome will be obtained from the National Health Insurance database.

Inpatient and outpatient diagnosis files during the 12-month period before study entry will be used to ascertain patients’ comorbidities and to calculate Charlson comorbidity scores, according to the following ICD-9-CM codes.

| Liver cirrhosis | 571.2, 571.5, 571.6 |
| --- | --- |
| Dialysis | ICD-9-CM codes: V451, V560, V568  Health insurance reimbursement codes: 58001C, 58001CA, 58002C, 58002CB, 58009B, 58010A, 58010B, 58011A, 58011AB, 58011B, 58011C, 58012A, 58012B, 58013C, 58014C, 58017B, 58017C, 58018C, 58019C, 58020C, 58021C, 58022C, 58023C, 58024C, 58025C, 58026C, 58027C, 58028C, 58029C, 58030B, 69006C, 69006C, CGDW1960012C, CGS01440042C, CGS01440132C, CKD006752L2X, CKD006753L2X, CKDD1135132C, CKDD1170052C, CKDD117005KD, CKDD1170092C, CKDD117009KD, CKDD122334BQ, CKDD124334BQ, CKDD1390032C, CKDD139003KD, CKDD154334BQ, CKDD1881CK2C, CKDD1881SK2C, CKDD1883CK2C, CKDD1883SK2C, CKDD210598SB, CKDD2140012C, CKDD214001KD, CKDD2140022C, CKDD214002KD, CKDD214554NL, CKDD2330742C, CKDD2420052C, CKDD242005KD, CKDD2450072C, CKDD245007KD, CKDD245540SB, CKDD245542SB, CKDD2480012C, CKDD257331BA, CKDD2690012C, CKDD269001KD, CKDD269033BA, CKDD2881452C, CKDD288155KD, CKDD2SL12PM0, CKDD2SL18PM0, CKDD323334BQ, CKDD3456032C, CKDD345603KD, CKP010T2022C, CKP018812N2C, CKP018812NKD, CKP018814N2C, CKP018814NKD, CKP018817N2C, CKP018817NKD, CKP018880N2C, CKP018880NKD, CKP018884N2C, CKP018888N2C, CKP018890N2C, CKP018890NKD, CKP022C410BT, CKP0413836FA, CKP04C4407BT, CKP04C4479BT, CKP04C8303BT, CPC0260048DV, FUK0500M60GA, FUK050M100GA, FUK05ST100GA, FUK05ST60PGA, FUK05TS404FN, FUK05TS413FN, HEF03PRFPEGA, HEF03PRLPEGA, HEF03PRTPEGA |
| Hypertension | 401-404 |
| Diabetes mellitus | 250 |
| Ischemic heart disease | 411, 413, 414 |
| Myocardial infarction | 410, 412 |
| Cardiac dysrhythmia/atrial fibrillation | 427 |
| Congestive heart failure | 428, 398.91, 402.01, 402.11, 402.91, 404.01, 404.11, 404.91, 404.03, 404.13, 404.93 |
| Stroke | 430, 431, 432, 433, 434, 436 |
| Peripheral vascular disease | 440.2, 440.4, 443.81, 443.9 |
| Disorders of lipid metabolism | 272 |
| Chronic lung disease | 490-496, 500-508 |
| Autoimmune disease | 710.2, 696.0, 696.1, 714.0, 710.0, 710.1, 710.4, 283.0, 245.2, 340, 358.0, 364.0, 364.3, 555, 556 |
| Dementia | 290.0-290.4, 291.2, 294.1, 331.0-331.2, 290.10-290.13, 290.20, 290.21, 290.40-290.43, 294.10, 294.11, 331.11, 331.19, 331.82 |
| Cancer | 140-208 |
| Human immunodeficiency virus infection | 042 |
| Opioid dependence or abuse | 304.0, 304.7, 305.5 |
| Peptic ulcer disease | 531-535, 578.0, 578.1, 578.9 |

**Outcome and follow-up plan**

**Primary outcome:** the first hospitalization due to any pre-specified infectious episodes ascertained from the National Health Insurance Database after study initiation.

Infection hospitalizations will be further classified according to specific site of infection, including septicemia, lower respiratory tract, intra-abdominal, reproductive and urinary tract, skin and soft tissue, osteomyelitis, necrotizing fasciitis, and infectious intestinal diseases, defined by the following ICD-9-CM codes.

| Infection syndrome | ICD-9-CM codes |
| --- | --- |
| Septicemia | 038, 041.9, 790.7, 785.52 |
| Lower respiratory tract infection | 480, 487, 481, 482, 483, 485, 486, 510, 513 |
| Intra-abdominal infection | 540, 541, 542, 562.01, 562.03, 562.11, 562.13, 566, 567, 569.5, 572.0, 572.1, 575.0 |
| Reproductive and urinary tract infection | 590, 599.0, 601, 604, 614, 615, 616 |
| Skin and soft tissue infection | 680, 681, 682, 683, 684, 685, 686 |
| Osteomyelitis | 711.0, 730 |
| Necrotizing fasciitis | 728.86 |
| Infectious intestinal diseases | 001-009 |

The patients can have more than one specific site of infection in their first infection hospitalization. However, they will no longer contribute person-time experiences after their first hospitalization due to infection.

Participants will be followed from health screening date till first hospitalized infection, death (based on vital registry), or the end of 2015, whichever occurred first.

**Secondary outcome**: infection related mortality.

The vital status and date of death for the study participants will be ascertained by linkage through the National Death Registry with a unique identification number.

Infection-related death will be defined by the death certificates codes according to the ICD-9 and ICD-10.

Participants will be followed from health screening date till death (based on vital registry), or the end of 2015, whichever occurred first.

**Statistical analysis**

The frequency distribution of baseline characteristics as well as Charlson comorbidity score among patients in five groups of liver disease categories will be tabulated (🡪 Table 1).

Crude incidence rates of hospitalization for any infection, specific site of infection, and infection-related mortality, along with 95% confidence interval using poisson distribution, will be calculated (🡪 Table 2).

Cox proportional regression models will be used to estimate the hazard ratios (HRs) and 95% confidence intervals of these infections for different liver disease categories, as compared with those without hepatitis B nor C and with normal liver function test results (🡪 Table 3).

Stratified analyses (🡪 Table 4) will be performed in hepatitis C patients according to

- the extent of ALT elevation (<1.5x UNL or ≥ 1.5x UNL)
- aspartate aminotransferase-to-platelet ratio index (APRI) (< or ≥ median APRI level)
- alcohol use (never or ever alcohol use).

Subgroup analysis will also be performed to investigate whether the risk may be modified by

- age (<50 and ≥50 years old)
- sex (men and women).

Potential effect medication will be assessed by observing overlap of the 95% confidence intervals in subgroups and a formal test for interaction.

Sensitivity analyses excluding the participants with following diagnoses will be performed to investigate the robustness of the results (🡪 Table 5):

- human immunodeficiency virus infection
- opioid dependence or abuse
