# Supplementary material for: Incidence of hospitalization for infection among patients with hepatitis B or C virus infection without cirrhosis in Taiwan: A cohort study
Source: PLoS Med. 2019 Sep 13;16(9):e1002894. doi: 10.1371/journal.pmed.1002894 (PMC6743759; doi:10.1371/journal.pmed.1002894)
Supplement: S2 Text — Checklist of items that should be included in reports of cohort studies. (DOC) [file pmed.1002894.s018.DOC]

STROBE Statement—Checklist of items that should be included in reports of ***cohort studies***

|  | Item No | Recommendation |
| --- | --- | --- |
| **Title and abstract** | 1 | (*a*) Indicate the study’s design with a commonly used term in the title or the abstract  **Abstract: Paragraph 2 *(Methods and Findings)*** |
|  |  | (*b*) Provide in the abstract an informative and balanced summary of what was done and what was found  **Abstract: Paragraph 1-3** |
| Introduction | | |
| Background/rationale | 2 | Explain the scientific background and rationale for the investigation being reported  **Introduction: Paragraph 1-3** |
| Objectives | 3 | State specific objectives, including any prespecified hypotheses  **Introduction, Paragraph 3** |
| Methods | | |
| Study design | 4 | Present key elements of study design early in the paper  **Methods:**  ***Risks for infectious disease–related morbidity and mortality among patients with NC-HBV or NC-HCV***  Data source and study population  ***Evaluate the impact of anti-HCV treatment on the risks of infectious diseases morbidity and mortality***  Paragraph 1. |
| Setting | 5 | Describe the setting, locations, and relevant dates, including periods of recruitment, exposure, follow-up, and data collection  **Methods:**  ***Risks for infectious disease–related morbidity and mortality among patients with NC-HBV or NC-HCV***  Data source and study population; Measurement of liver disease status and other covariates; Outcome and follow-up plan.  ***Evaluate the impact of anti-HCV treatment on the risks of infectious diseases morbidity and mortality,***  Paragrah 1and 2; Study drugs and study cohort assembly; Follow-up and outcomes; covariate aassessment. |
| Participants | 6 | (*a*) Give the eligibility criteria, and the sources and methods of selection of participants. Describe methods of follow-up  **Methods:**  ***Risks for infectious disease–related morbidity and mortality among patients with NC-HBV or NC-HCV***  Data source and study population; Measurement of liver disease status and other covariates; Outcome and follow-up plan; Figure 1.  ***Evaluate the impact of anti-HCV treatment on the risks of infectious diseases morbidity and mortality,***  Paragrah 1and 2; Study drugs and study cohort assembly; Follow-up and outcomes; covariate aassessment; Figure 3. |
|  |  | (b) For matched studies, give matching criteria and number of exposed and unexposed  **Methods:**  ***Evaluate the impact of anti-HCV treatment on the risks of infectious diseases morbidity and mortality,***  Statistical analyses and Figure 3. |
| Variables | 7 | Clearly define all outcomes, exposures, predictors, potential confounders, and effect modifiers. Give diagnostic criteria, if applicable  **Methods:**  ***Risks for infectious disease–related morbidity and mortality among patients with NC-HBV or NC-HCV***  Measurement of liver disease status and other covariates, Outcome and follow-up plan and Statistical analysis.  ***Evaluate the impact of anti-HCV treatment on the risks of infectious diseases morbidity and mortality,***  Study drugs and study cohort assembly; Follow-up and outcomes; covariate aassessment; Statistical analyses; Auxillary analyses.  **See also: Supplementary Table 1.** |
| Data sources/ measurement | 8 | For each variable of interest, give sources of data and details of methods of assessment (measurement). Describe comparability of assessment methods if there is more than one group  **Methods:**  ***Risks for infectious disease–related morbidity and mortality among patients with NC-HBV or NC-HCV***  Measurement of liver disease status and other covariates, Outcome and follow-up plan and Statistical analysis.  ***Evaluate the impact of anti-HCV treatment on the risks of infectious diseases morbidity and mortality,***  Study drugs and study cohort assembly; Follow-up and outcomes; covariate aassessment; Statistical analyses; Auxillary analyses. |
| Bias | 9 | Describe any efforts to address potential sources of bias  **Methods:**  ***Risks for infectious disease–related morbidity and mortality among patients with NC-HBV or NC-HCV***  Statistical analysis.  ***Evaluate the impact of anti-HCV treatment on the risks of infectious diseases morbidity and mortality,***  Statistical analyses and Auxillary analyses. |
| Study size | 10 | Explain how the study size was arrived at  **Methods:**  ***Risks for infectious disease–related morbidity and mortality among patients with NC-HBV or NC-HCV***  Data source and study design (nb. no pre-defined study size, all eligible individuals included)  ***Evaluate the impact of anti-HCV treatment on the risks of infectious diseases morbidity and mortality,***  Paragraph 2 (nb. no pre-defined study size, all eligible individuals included). |
| Quantitative variables | 11 | Explain how quantitative variables were handled in the analyses. If applicable, describe which groupings were chosen and why  **Methods:**  ***Risks for infectious disease–related morbidity and mortality among patients with NC-HBV or NC-HCV***  Measurement of liver disease status and other covariates, and Statistical analysis.  ***Evaluate the impact of anti-HCV treatment on the risks of infectious diseases morbidity and mortality,***  Covariate assessment. |
| Statistical methods | 12 | (*a*) Describe all statistical methods, including those used to control for confounding  **Methods:**  ***Risks for infectious disease–related morbidity and mortality among patients with NC-HBV or NC-HCV***  Statistical analysis.  ***Evaluate the impact of anti-HCV treatment on the risks of infectious diseases morbidity and mortality,***  Statistical analyses and Auxillary analyses. |
|  |  | (*b*) Describe any methods used to examine subgroups and interactions  **Methods:**  ***Risks for infectious disease–related morbidity and mortality among patients with NC-HBV or NC-HCV***  Statistical analysis. |
|  |  | (*c*) Explain how missing data were addressed  **Methods:**  ***Risks for infectious disease–related morbidity and mortality among patients with HCV and without cirrhosis***  Data source and study population; Supplementary Table 2. |
|  |  | (*d*) If applicable, explain how loss to follow-up was addressed  **Methods:**  ***Risks for infectious disease–related morbidity and mortality among patients with NC-HBV or NC-HCV***  Data source and study population  ***Evaluate the impact of anti-HCV treatment on the risks of infectious diseases morbidity and mortality,***  Paragraph 2. |
|  |  | (*e*) Describe any sensitivity analyses  **Methods:**  ***Risks for infectious disease–related morbidity and mortality among patients with NC-HBV or NC-HCV***  Statistical analysis.  ***Evaluate the impact of anti-HCV treatment on the risks of infectious diseases morbidity and mortality,***  Auxillary analyses**.** |
| Results | | |
| Participants | 13 | (a) Report numbers of individuals at each stage of study—eg numbers potentially eligible, examined for eligibility, confirmed eligible, included in the study, completing follow-up, and analysed  **Results:**  ***Risks for infectious disease–related morbidity and mortality among patients with NC-HBV or NC-HCV***  Paragraph 1 and Figure 1.  ***Evaluate the impact of anti-HCV treatment on the risks for infectious diseases morbidity and mortality***  *The impact of HCV antiviral treatment on infection risks,* Paragraph 1;  *The impact of HBV antiviral treatment on infection risks,* paragraph 1;  Figure 4. |
|  |  | (b) Give reasons for non-participation at each stage  **Results: Figure 1 and Figure 4.** |
|  |  | (c) Consider use of a flow diagram  **Figure 1 and Figure 4.** |
| Descriptive data | 14 | (a) Give characteristics of study participants (eg demographic, clinical, social) and information on exposures and potential confounders  **Results:**  ***Risks for infectious disease–related morbidity and mortality among patients with NC-HBV or NC-HCV***  Paragraph 1 and 2; Table 1.  ***Evaluate the impact of anti-HCV treatment on the risks for infectious diseases morbidity and mortality***  *The impact of HCV antiviral treatment on infection risks,* Paragraph 2 and 3; S12 Table.  *The impact of HBV antiviral treatment on infection risks,* Paragraph 1; S14 Table.. |
|  |  | (b) Indicate number of participants with missing data for each variable of interest  **Not applicable.** *(Participants with missing data were excluded from the analysis.)* |
|  |  | (c) Summarise follow-up time (eg, average and total amount)  **Results:**  ***Risks for infectious disease–related morbidity and mortality among patients with NC-HBV or NC-HCV***  Paragraph 3;Table 2.  ***Evaluate the impact of anti-HCV treatment on the risks for infectious diseases morbidity and mortality***  *The impact of HCV antiviral treatment on infection risks,* Paragraph 5; S13 Table.  *The impact of HBV antiviral treatment on infection risks,* Paragraph 2; S15 Table. |
| Outcome data | 15 | Report numbers of outcome events or summary measures over time  **Results:**  ***Risks for infectious disease–related morbidity and mortality among patients with NC-HBV or NC-HCV***  Paragraph 3 and 4;Table 2.  ***Evaluate the impact of anti-HCV treatment on the risks for infectious diseases morbidity and mortality***  *The impact of HCV antiviral treatment on infection risks,* Paragraph 5; S13 Table.  *The impact of HBV antiviral treatment on infection risks,* Paragraph 2; S15 Table. |
| Main results | 16 | (*a*) Give unadjusted estimates and, if applicable, confounder-adjusted estimates and their precision (eg, 95% confidence interval). Make clear which confounders were adjusted for and why they were included  **Results:**  ***Risks for infectious disease–related morbidity and mortality among patients with NC-HBV or NC-HCV***  Paragraph 5;Table 3.  ***Evaluate the impact of anti-HCV treatment on the risks for infectious diseases morbidity and mortality***  *The impact of HCV antiviral treatment on infection risks,* Paragraph 6; Table 4.  *The impact* of *HBV antiviral treatment on infection risks,* Paragraph 3; S16 Table. |
|  |  | (b) Report category boundaries when continuous variables were categorized  **Table 1, 3 and S4-11 Tables.** |
|  |  | (c) If relevant, consider translating estimates of relative risk into absolute risk for a meaningful time period  **Results:**  ***Risks for infectious disease–related morbidity and mortality among patients with NC-HBV or NC-HCV***  Paragraph 3 and 4;Table 2.  ***Evaluate the impact of anti-HCV treatment on the risks for infectious diseases morbidity and mortality***  *The impact of HCV antiviral treatment on infection risks,* Paragraph 5; S13 Table.  *The* impact *of HBV antiviral treatment on infection risks,* Paragraph 2; S15 Table. |
| Other analyses | 17 | Report other analyses done—eg analyses of subgroups and interactions, and sensitivity analyses  **Results:**  ***Risks for infectious disease–related morbidity and mortality among patients with NC-HBV or NC-HCV***  Paragraph 5-7; S4-11 Tables.  ***Evaluate the impact of anti-HCV treatment on the risks for infectious diseases morbidity and mortality***  *The impact of HCV antiviral treatment on infection risks,* Paragraph 6; Table 4.  *The impact of HBV antiviral treatment on infection risks,* Paragraph 3; S16 Table. |
| Discussion | | |
| Key results | 18 | Summarise key results with reference to study objectives  **Discussion: Paragraph 1** |
| Limitations | 19 | Discuss limitations of the study, taking into account sources of potential bias or imprecision. Discuss both direction and magnitude of any potential bias  **Discussion: Paragraph 8** |
| Interpretation | 20 | Give a cautious overall interpretation of results considering objectives, limitations, multiplicity of analyses, results from similar studies, and other relevant evidence  **Discussion: Paragraph 2-7.** |
| Generalisability | 21 | Discuss the generalisability (external validity) of the study results  **Discussion: paragraph 8.** |
| Other information | | |
| Funding | 22 | Give the source of funding and the role of the funders for the present study and, if applicable, for the original study on which the present article is based  **The issue has been stated in the electronic submission system.** |

*Give information separately for exposed and unexposed groups.

**Note:** An Explanation and Elaboration article discusses each checklist item and gives methodological background and published examples of transparent reporting. The STROBE checklist is best used in conjunction with this article (freely available on the Web sites of PLoS Medicine at http://www.plosmedicine.org/, Annals of Internal Medicine at http://www.annals.org/, and Epidemiology at http://www.epidem.com/). Information on the STROBE Initiative is available at http://www.strobe-statement.org.
